# Unraveling the Microscopic Mechanism of Molecular Ion Interaction with Monoclonal Antibodies: Impact on Protein Aggregation

**DOI:** 10.1021/acs.molpharmaceut.3c00963

**Published:** 2024-02-12

**Authors:** Suman Saurabh, Qinkun Zhang, John M. Seddon, Jian R. Lu, Cavan Kalonia, Fernando Bresme

**Affiliations:** † Department of Chemistry, Molecular Sciences Research Hub, Imperial College, London W12 0BZ, U.K.; ‡ Biological Physics Group, School of Physics and Astronomy, Faculty of Science and Engineering, The University of Manchester, Oxford Road, Manchester M13 9PL, U.K.; § Dosage Form Design and Development, BioPharmaceutical Development, BioPharmaceuticals R&D, AstraZeneca, Gaithersburg, Maryland 20878, United States

**Keywords:** monoclonal antibody, aggregation, buffer, citrate, phosphate, histidine, debye length, ionic bridge

## Abstract

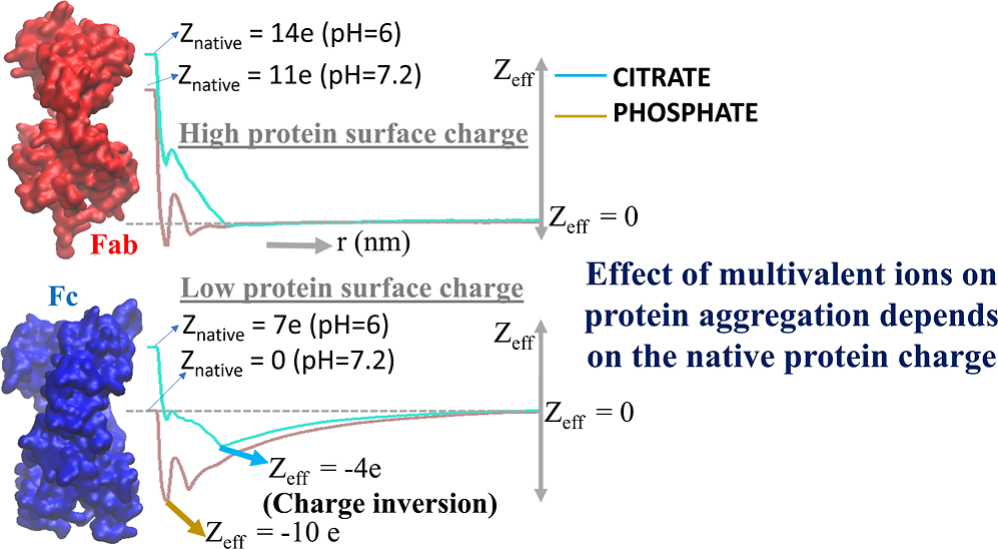

Understanding and predicting protein aggregation represents one of the major challenges in accelerating the pharmaceutical development of protein therapeutics. In addition to maintaining the solution pH, buffers influence both monoclonal antibody (mAb) aggregation in solution and the aggregation mechanisms since the latter depend on the protein charge. Molecular-level insight is necessary to understand the relationship between the buffer–mAb interaction and mAb aggregation. Here, we use all-atom molecular dynamics simulations to investigate the interaction of phosphate (*Phos*) and citrate (*Cit*) buffer ions with the Fab and Fc domains of mAb COE3. We demonstrate that *Phos* and *Cit* ions feature binding mechanisms, with the protein that are very different from those reported previously for histidine *(His)*. These differences are reflected in distinctive ion-protein binding modes and adsorption/desorption kinetics of the buffer molecules from the mAb surface and result in dissimilar effects of these buffer species on mAb aggregation. While *His* shows significant affinity toward hydrophobic amino acids on the protein surface, *Phos* and *Cit* ions preferentially bind to charged amino acids. We also show that *Phos* and *Cit* anions provide bridging contacts between basic amino acids in neighboring proteins. The implications of such contacts and their connection to mAb aggregation in therapeutic formulations are discussed.

## 1 Introduction

Monoclonal antibodies (mAbs) have gained relevance in treating cancer, autoimmune diseases, infectious diseases, and metabolic disorders.^1,2^ Therapeutic formulations used in subcutaneous administration, relevant in chronic conditions like arthritis, require formulations with a high concentration of mAb, typically >100 mg/mL, to ensure suitable delivery volumes. Under these conditions, mAbs are prone to aggregation, which could reduce their maximum shelf life. In addition, aggregate levels need to be controlled, as high levels of aggregates could potentially trigger an immune response.^3−6^ The need to solve the aggregation problem drives significant efforts in search of low-cost approaches to assist formulation development.^7−10^

The pH of mAb formulations is an important stability-determining factor in the preparation of formulations for medical applications since the pH regulates the protein charge. Therefore, buffers such as histidine (*His*), phosphate (*Phos*), acetate, citrate (*Cit*), aspartate, and tris, among many others, are often employed in protein formulations to maintain the solution pH.^11−15^ Most mAb formulations include one of the buffers listed above. Interestingly, at high mAb concentrations, some formulations feature self-buffering, with the buffer action being performed by the mAb itself.^11^

Experimental studies demonstrated that buffer molecules influence the solution stability by modifying protein–protein interaction by binding to the protein surface, leading to either an increase or a decrease of the solution stability.^16−19^ Furthermore, buffer molecules might interact with other components in a formulation, possibly impacting formulation stability.^20^ The role of buffers on solution stability has been linked to how buffer ions influence the secondary structure of mAbs and to the specific binding of buffer ions to aggregation-prone regions on the mAb surface. However, the microscopic mechanisms associated with buffers that influence the stability of mAb formulations are still under debate.^19^

*His*, *Phos*, and *Cit* are among the most widely used buffers in therapeutic formulations.^16^*His* is known to stabilize mAbs against aggregation.^19,21,22^ The stabilizing mechanism appears to be uncorrelated with *His*’s role in preserving the mAb secondary structure.^23^ However, there is evidence that the stability of the formulations increases with *His* concentration.^23^ Dynamic light scattering (DLS) experiments indicate that the hydrodynamic radius of mAbs in solution depends on *His* concentration, an observation that was rationalized considering the impact of the *His*–mAb interaction on mAb flexibility. It was also shown that increasing the NaCl concentration at a constant, *His* concentration did not have any effect on the hydrodynamic radius of the mAb. This result may indicate that the ionic strength does not affect the *His*–mAb interaction.^24^ However, in another experimental study, Kalonia et al.^19^ found that NaCl does affect the degradation rate of therapeutic formulations stabilized with *His*. We investigated in an earlier work the interaction between *His* and the Fab/Fc fragments of the mAb COE3.^25^ Using molecular dynamics (MD) simulations, we concluded that the stabilizing effect of *His* on mAb formulations originates from its interaction with surface-exposed hydrophobic amino acid residues.

Experimental studies showed that the *Phos* and *Cit* buffers are less effective than *His* in preventing mAb aggregation.^16^ Kalonia et al.^19^ concluded from solubility measurements and aggregation data of a IgG1 mAb, performed using elevated temperature conditions, that the *His* buffer provides better stability against aggregation than *Cit* at pH values of 4.5 and 6.5. The second virial coefficient extracted from the static light scattering (SLS) measurements of mAb formulations containing *His* indicates that the mAb–mAb interaction is repulsive, while the interaction is attractive in the presence of *Cit*. This observation agrees with the work of Barnett et al.,^17^ who performed size exclusion chromatography (SEC) and SLS experiments of antistreptavidin IgG1 in *Cit* solutions.

Joshi et al.^26^ investigated the behavior of IgG1 mAbs in solutions containing different buffers. They used SEC and circular dichroism experiments to show that the *Cit* buffer resulted in stronger mAb aggregation than acetate, glycine, and tris. Demeule et al.^27^ reported the formation of μm-sized IgG1 mAb aggregates in *Phos* buffer and found that the aggregation correlates with the conformational changes in the protein around hydrophobic amino acids. This observation suggests an aggregation mechanism connected to the perturbation of the protein secondary structure. Brudar and Hribar-Lee^28^ reported a negative virial coefficient for solutions of hen egg-white lysozyme in *Phos* buffer. The effect was attributed to electrostatic screening of the surface charge of the protein by *Phos* ions. All these experimental studies point toward a less effective stabilizing and, in some cases, destabilizing effect of *Cit* and *Phos* buffer as compared to *His*.

The contrasting effects on mAb solution stability of *His*, *Phos*, and *Cit* buffers provide an important basis for understanding the microscopic mechanisms underlying the effect of buffers on mAb aggregation based on the different interaction modes of these buffer ions with the mAb surface. Determination of the underlying molecular mechanisms will help in the design of optimum formulations and assist protein engineering efforts aimed at designing proteins with surfaces that are more resistant to aggregation. In this work, we perform all-atom MD simulations of Fab and Fc fragments of mAb COE3 in aqueous solutions containing *Phos* and *Cit* buffers. We use these simulations to highlight microscopic details associated with specific protein–buffer interactions. We discuss the impact of these different interaction modes on the effective inter-fragment interaction and the potential role of these interactions in solution stability. Furthermore, we compare the results obtained with *Cit* and *Phos* with our previously reported data on *His*–mAb interaction.^25^

## 2 Materials and Methods

### 2.1 Molecular Dynamics of Protein and Buffer Solutions

#### 2.1.1 Models of Fab and Fc Fragments

The Fab and Fc fragments used in this study are part of the monoclonal antibody COE3. The Fc fragment of the antibody has a 100% sequence similarity to the Fc domain of the anti-HIV1 human IgG B12 (PDB id: 1HZH).^29^ The Fab domain has a 73% similarity to the 1HZH Fab.^25^ The initial model of the Fc fragment was obtained by deleting the two Fabs from the structure of 1HZH. The Fc fragment consists of the C-terminal halves of the two heavy chains of the mAb. The Fc structure contains six disulfide bonds, of which two are interchain bonds in the hinge region, while the remaining four are intrachain bonds. To build the Fab fragment, we followed the work by Singh et al.^30^ The Fab fragment consists of the mAb light chain and the N-terminal half of one of the heavy chains of the mAb. The Fab structure contains five disulfide bonds (one interchain and four intrachain).

#### 2.1.2 Determination of the Protein Charge

The simulations with *Phos* buffer were performed at pH 7.2, while those with *Cit* buffer were performed at pH 6. These pH values fall in the typical range used in experimental studies employing these two buffers. We compare our results to simulations of the same mAb fragments performed with the *His* buffer. Those simulations were performed at the relevant experimental pH = 6 in the presence of 20 mM *His*, which amounted to 20 *His* molecules (10 in the +1 charge state and 10 neutral *His* molecules).^25^ The protonation state of the titratable residues of the proteins at the specific pH was calculated using the propKa3.1 methodology.^31^ At pH 7.2, Fab has a net charge of +11*e*, while the Fc fragment is neutral. At pH 6, the Fc domain has a charge of +7*e*, and the Fab domain has a charge of +14*e*. The stronger dependence of the charge of the Fc fragment on pH emerges from the presence of a larger number of *His* residues (12) in the Fc sequence compared to Fab (5).

#### 2.1.3 Buffer Protonation States

The *Phos* buffer simulations were performed at a pH = 7.2 with a buffer concentration of 20 mM, close to the typical concentration used in mAb formulations.^32^

At pH 7.2, the *Phos* ion predominantly exists in 2 charge states, −2*e* and −1*e* (see Figure S1 of Supporting Information). The fraction of different buffer charge states was calculated using the Henderson–Hasselbalch (HH) equation^33,34^
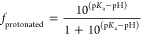
1

At pH = 7.2, half of the *Phos* molecules will be in the −2*e* charged state.

Similarly, the *Cit* ions in our simulations were present in two different charge states, −2*e* and −3*e*. The HH equation predicts a 4:3 ratio for the −2*e*/–3*e* states at pH 6, the pH employed in the *Cit* buffer simulations. Table S1 of the Supporting Information contains information on the number of buffer ions and water molecules employed in our computations.

### 2.2 Simulation Systems

#### 2.2.1 Single Protein

**Figure 1:**
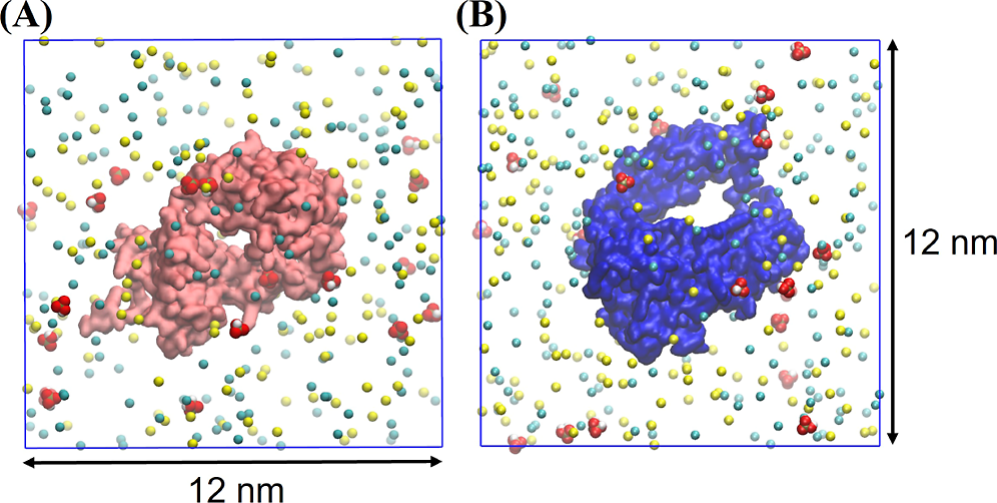
Initial system configurations for the (A) Fab and (B) Fc fragments in *Phos* buffer. Oxygens in the *Phos* anion are shown in red. Na^+^ ions are shown in cyan and the Cl^–^ ions are in yellow. Water molecules are not shown for clarity.

The Fab/Fc fragment with charges corresponding to pH 6 and 7.2 was placed at the center of a cubic periodic box of side 12 nm. The *Phos* and *Cit* ions were then randomly added around the protein using the gmx insert-molecules tool of GROMACS,^35,36^ which ensures a homogeneous distribution of the ions in the box. The boxes were filled with 20 mM of *Cit* ions (pH = 6) or *Phos* ions (pH = 7.2) (see Section 2.1.3 for information on the buffer protonation states). The systems were solvated with 3-point water and neutralized by adding Na^+^ ions. Additional Na^+^ and Cl^–^ ions were added to achieve a salt concentration (150 mM) similar to the physiological condition. The initial system configurations for the Fab and Fc fragments in *Phos* buffer are shown in Figure 1. In addition, we performed simulations in the absence of NaCl to assess the impact of solution composition on the adsorption of the buffer ions on the protein surface. All of the simulations were performed with the TIPs3P water model and the CHARMM36m force field (ff) for the ions and amino acids. CHARMM36m compatible *Phos* and *Cit* ion parameters were generated using Cgenff.^37,38^

#### 2.2.2 Two Protein Systems

To study the binding of buffer ions to protein–protein interfaces and the effect of buffer on the interaction between mAb fragments, we performed simulations of 2 protein systems.

**Figure 2:**
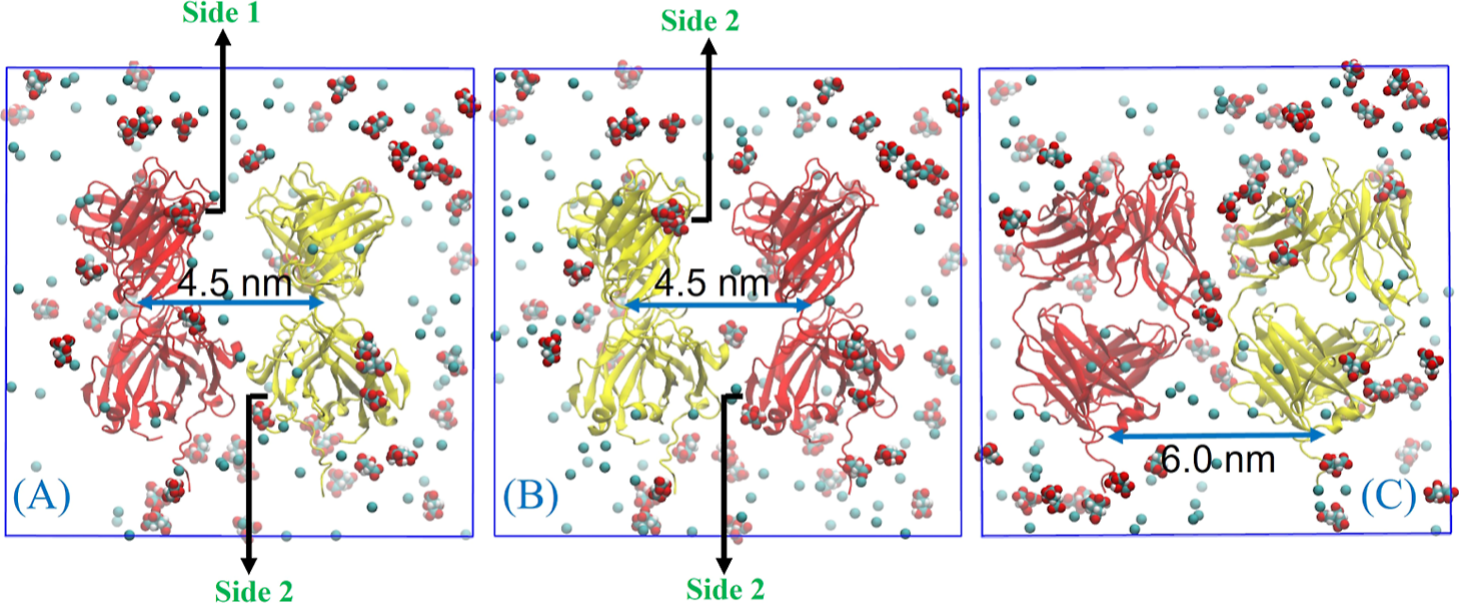
Initial configurations used to generate three independent simulation trajectories of the two Fab system. Configurations show two Fab fragments in the presence of *Cit* buffer (red–cyan–white spheres). Similar simulations were performed with *Phos* buffer. Numbers indicate the initial center of mass distance between the proteins. For the Fab fragment, side 1 and side 2 refer to two different flat faces of the Fab fragment that differ in the net charge. Side 2 has a larger net positive charge as compared to side 1. Charged amino acid composition of the two faces is shown in Figure S4 of the Supporting Information. Simulations with similar starting conformations were performed for the 2-Fc and the Fab–Fc systems as well.

Two mAb fragments (Fab–Fab, Fc–Fc, and Fab–Fc) with charges corresponding to pH 6 and 7.2 were placed at a center-to-center distance of 4.5 nm (see Figure 2A,B) or 6 nm (see Figure 2C). The protein pairs were then placed at the center of a cubic periodic box of side 12 nm. The boxes were then filled with 50 mM equivalent of *Cit* ions (pH 6) or *Phos* ions (pH = 7.2). The boxes were then solvated with TIPs3P water and neutralized by adding Na^+^ ions. In addition, we performed the simulations without a buffer but kept the ionic strength fixed. The ionic strength was maintained by replacing the buffer ions with an equivalent number of Cl^–^ ions. Details of all of the simulations performed in this work are provided in Table 1.

**Table 1:** Summary of the Systems Simulated in This Work^a^

system	system name	system details	pH	buffer concn (mM)	NaCl concn (mM)	time (ns)
1	Fab_*Phos*_^7.2^	Fab with *Phos*	7.2	20	150	200 × 5
2	Fc_*Phos*_^7.2^	Fc with *Phos*	7.2	20	150	200 × 5
3	Fab_*Phos*_^7.2^-ns	Fab with *Phos*	7.2	20	0	200 × 3
4	Fc_*Phos*_^7.2^-ns	Fc with *Phos*	7.2	20	0	200 × 3
5	Fab_*Cit*_^6^	Fab with *Cit*	6.0	20	150	200 × 5
6	Fc_*Cit*_^6^	Fc with *Cit*	6.0	20	150	200 × 5
7	Fab_*Cit*_^6^-ns	Fab with *Cit*	6.0	20	0	200 × 3
8	Fc_*Cit*_^6^-ns	Fc with *Cit*	6.0	20	0	200 × 3
9	2Fab_*Cit*_^6^	2 Fabs with *Cit*	6.0	50	0	200 × 3
10	2Fab_*Phos*_^7.2^	2 Fabs with *Phos*	7.2	50	0	200 × 3
11	2Fab_*nb*_^6^	2 Fabs with no buffer	6.0	0	90	200 × 3
12	2Fab_*nb*_^7.2^	2 Fabs with no buffer	7.2	0	50	200 × 3
13	2Fc_*Cit*_^6^	2 Fcs with *Cit*	6.0	50	0	200 × 3
14	2Fc_*Phos*_^7.2^	2 Fcs with *Phos*	7.2	50	0	200 × 3
15	2Fc_*nb*_^6^	2 Fcs with no buffer	6.0	0	108	200 × 3
16	2Fc_*nb*_^7.2^	2 Fcs with no buffer	7.2	0	76	200 × 3
17	(Fab–Fc)_*Cit*_^6^	Fab and Fc with *Cit*	6.0	50	0	200 × 3
18	(Fab–Fc)_*Phos*_^7.2^	Fab and Fc with *Phos*	7.2	50	0	200 × 3
19	(Fab–Fc)_*nb*_^6^	Fab and Fc with no buffer	6.0	0	100	200 × 3
20	(Fab–Fc)_*nb*_^7.2^	Fab and Fc with no buffer	7.2	0	65	200 × 3

a See Materials and Methods section for details on the charge of the proteins and the charge state composition of the *Phos* and *Cit* ions. Multiple (5 or 3) independent simulations were performed for each system, starting from different initial positions of the buffer and Na^+^/Cl^–^ ions. The initial configuration for each trajectory was generated by randomly adding ions to the simulation box. “Time” indicates the simulation time for the production run. Subscripts “*Phos*”, “*Cit*”, and “*nb*” in the system name refer to phosphate, citrate, and no buffer conditions, respectively. The numbers in the superscript indicate the simulation pH. “ns” indicates a “no salt” condition (absence of NaCl). A total of 68 MD simulations with a cumulative simulation time of 13.6 μs were performed in this work.

### 2.3 Simulation Protocol

All the simulations reported in this work were performed using the GROMACS(2021.3) software^35,36^ package. The systems were first minimized by using the steepest descent method to remove bad contacts between the water molecules, ions, and atoms belonging to the protein. Following minimization, the systems were pre-equilibrated for 1 ns in the *NVT* ensemble at a temperature of 300 K, keeping the protein atoms harmonically restrained (*k* = 1000 kJ/mol/nm^2^) at their initial positions. The systems were then subjected to a 1 ns long unrestrained equilibration in the *NPT* ensemble at a constant temperature of 300 K and a pressure of 1 bar. Following equilibration, 200 ns long production runs were performed in the *NPT* ensemble. In all our simulations, the canonical *v*-rescale thermostat^39^ was used for temperature control with a temperature coupling constant of 0.5 ps. During equilibration, the Berendsen barostat^40^ was used, with a pressure coupling constant of 0.5 ps, while the Parrinello–Rahman barostat^41^ (coupling constant of 2.0 ps) was used for production runs.

For the 2-protein systems, the minimization and *NVT* pre-equilibration steps were performed, as discussed above. The systems were then equilibrated in the *NPT* ensemble for 20 ns, keeping the two proteins restrained to their initial position and conformation to allow the buffer ions to adsorb on the proteins. Following this, unrestrained, 200 ns long production runs were performed in the *NPT* ensemble.

The particle mesh Ewald^42^ method was used to compute the electrostatic interaction. We employed a cutoff of 1 nm for the dispersion interaction. Long-range pressure corrections were included. A simulation time step of 2 fs was employed, and the bonds involving hydrogens were held rigid using the LINCS algorithm.^43^

## 3 Results and Discussion

### 3.1 Buffer–Protein and Buffer–Ion Interaction

One of the questions we want to address in this work is the specificity of the buffer to adsorb on the protein surface. With this aim, we calculated the radial distribution function (rdf) of the buffer around the protein surface (from Fab_*Phos*_^7.2^, Fc_*Phos*_^7.2^, Fab_*Cit*_^6^, and Fc_*Cit*_^6^ simulations; see Table 1) using the gmx rdf -surf tool in GROMACS. The tool calculates the number of atoms (belonging to the buffer ions) in a subvolume (bin) at a specific distance, *r*, from the protein surface. The number of atoms in each bin is then divided by the bin width to calculate the rdf. Hence, the integral of this rdf gives the number of buffer atoms up to a distance of *r* from the protein surface.

**Figure 3:**
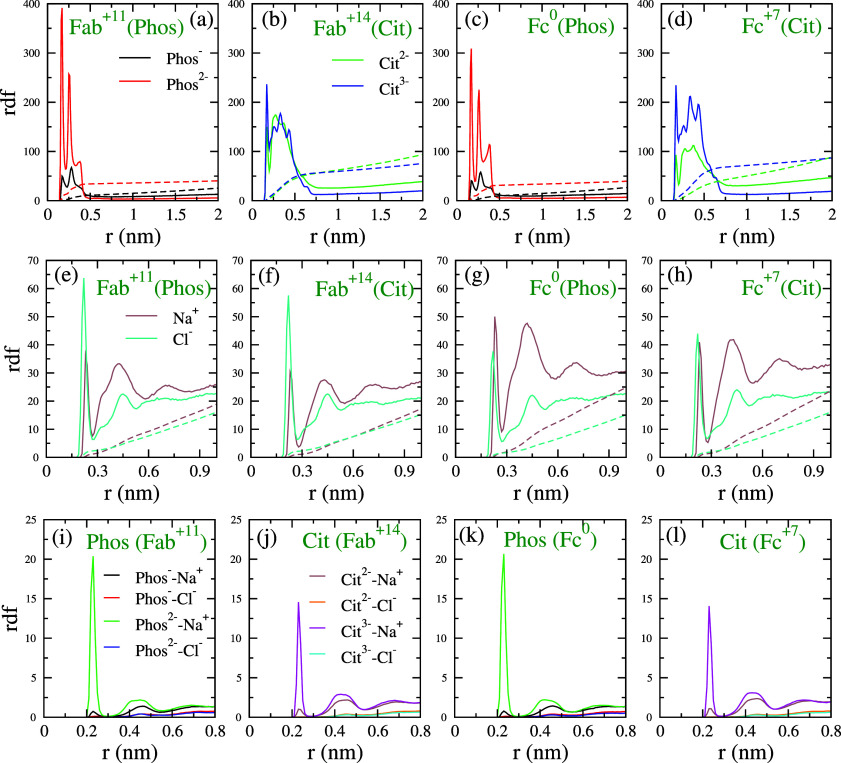
(a–d) Radial distribution of the atoms belonging to *Phos* and *Cit* ions around the Fab and Fc surface. (e–h) Radial distribution of Na^+^ and Cl^–^ ions around the Fab and Fc fragments for different systems. (i–l) Radial distribution of Na^+^ and Cl^–^ ions around the buffer ions. The rdf’s have been averaged over the last 190 ns of the five 200 ns long runs.

We find clear differences in the interaction of the different *Phos* anions with the Fab fragment. Higher charge (−2*e*) leads to stronger ionic layers, as shown by the height of the rdf peaks (cf. *Phos*^–^ and *Phos*^2–^ in Figure 3). Hence, we observe a higher affinity for *Phos*^2–^ than *Phos*^–^ to adsorb on either the Fc or the Fab surface. This observation points to a stronger electrostatic interaction for divalent *Phos*. Interestingly, the different charge states of the *Cit* ions feature similar adsorption behavior for Fab, with a slightly higher adsorption of *Cit*^3–^ than *Cit*^2–^. Given the higher charge of the *Cit*^3–^ ions, this result might appear surprising. The rdf shows that *Cit* ions are also larger, with the rdf peaks extending up to 0.75 nm instead of 0.45 nm, as in the case of *Phos*. The larger effective size of the *Cit* ions (and the resulting lower charge density) would lead to the weakening of the electrostatic interaction, which may explain the weaker dependence of ion adsorption with ion charge. Figure 3 also shows the rdf of Na^+^ and Cl^–^ ions around the Fab and Fc fragments. For the Fc fragment, which has charges of +7 and 0 at pH = 6 and 7.2, respectively, the adsorption of the Na^+^ and Cl^–^ ions is similar. For the Fab fragment (with charges of +14 and +11 at pH = 6 and 7.2, respectively), the Cl^–^ ions adsorb more strongly than the Na^+^ ions. These observations are in line with our earlier study,^44^ where we found stronger adsorption of Na^+^ ions on the Fc surface with the Charmm27 ff.

Overall, our simulations show that the adsorption of Cl^–^ is stronger on the highly charged Fab than on Fc (see Figure 3e–h). The adsorption of *Phos* follows a similar behavior (cf. Figure 3a–h). However, for *Cit*, the adsorption depends less strongly on the protein charge. These results point to an ion adsorption mechanism that is driven mostly by electrostatic interaction for small ions, while other factors determine the adsorption for larger ions, such as *Cit*, where the ionic charge is more delocalized. We will expand our analysis of the binding mechanism for these ions in Section 2.3 below.

We also calculated the rdf of the Na^+^ and Cl^–^ ions around the buffer ions. The Na^+^ ions interact preferentially with *Phos*^2–^ and *Cit*^3–^, highlighting the importance of the Coulombic interaction in determining ion-binding. However, the interaction with *Cit*^2–^ is weaker, as shown by the height of the main peak. This indicates that *Cit*^2–^ and Na^+^ form weaker ion pairs than *Cit*^3–^. The interaction of Na^+^ ions with *Phos*^2–^ is much stronger than the Na^+^–*Cit*^2–^ interaction, highlighting the relevance of the buffer charge density with respect to its electrostatic interaction with other components in solution.

We have focused so far on the structural aspects of the ionic distribution. We explore later in Section 3.5 how ion accumulation modifies the protein charge and potentially the inter-protein interaction. In the following, we compute a free energy map showing the buffer-adsorption landscape projected onto the protein surface. This analysis is aimed at identifying “hot spots” for the binding of buffer on the protein surface.

### 3.2 Free Energy of Ion Adsorption on the Proteins and Buffer Adsorption Index

To identify the prominent buffer-binding regions on the surface of Fab and Fc fragments as well as the relative importance of different surface residues with respect to buffer binding, we computed the relative residue-level free energy of buffer binding on the protein surface. For each of the five independent trajectories, the number of atomic contacts (*N*^*i*^) between each of the protein residues (*i*) and the *Phos*/*Cit* molecules was calculated. We define an atomic contact between a protein residue and a buffer molecule when the distance between any protein–buffer atomic pair is ≤0.4 nm. The number of atomic contacts between the residue and buffer molecules in a simulation frame is then calculated as the number of such atomic pairs and averaged over time to obtain the time averaged number of atomic contacts over the whole trajectory. We adopt here the same distance criterion we used before to investigate the adsorption of *His* on COE3.^25^ The calculations discussed below were averaged over five independent trajectories in order to calculate *N*_avg_^*i*^.

The buffer adsorption index (BAI)^25^
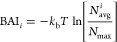
2quantifies the relative free energy of buffer-protein adsorption, for each amino acid residue (*i*) in the Fab or Fc fragment. *N*_max_ is the largest value among all *N*_avg_^*i*^, corresponding to the protein residue showing the highest affinity for buffer adsorption. We note that *N*_max_ is the overall highest number of contacts, irrespective of the buffer charge state. Hence, we use the same free energy origin for the different charge state of a givenbuffer species.

**Figure 4:**
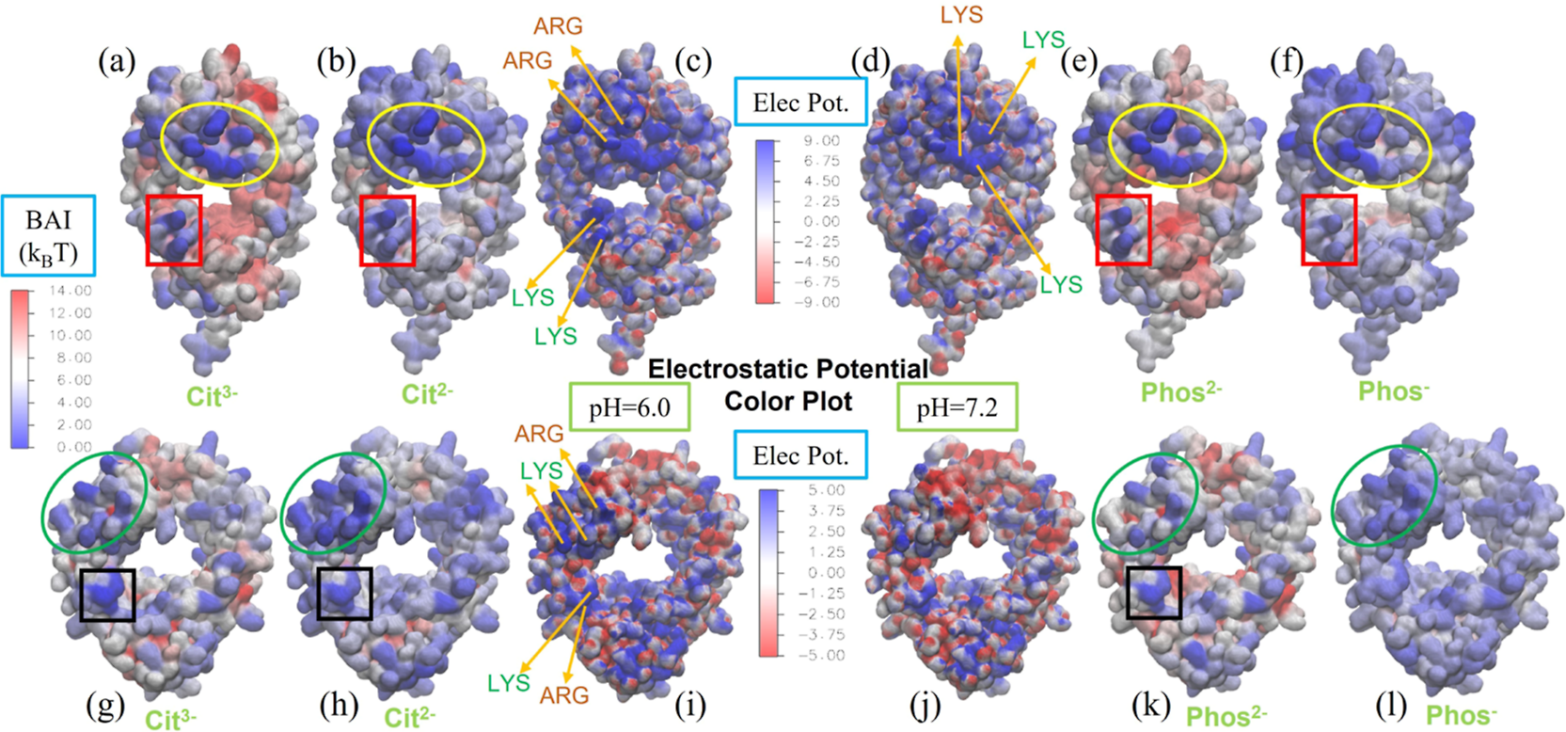
BAI obtained with eq 2 and represented as a color plot projected on the surface of Fab (top) and Fc (bottom) fragments, for (a,g) *Cit*^3–^, (b,h) *Cit*^2–^, (e,k) *Phos*^2–^, and (f,l) *Phos*^–^ buffer molecules. Lower BAI value for an amino acid residue corresponds to a higher number of contacts between the residue and the buffer molecules. We compare the BAI with the APBS electrostatic potential color plot at pH = 6 (c,i) and pH = 7.2 (d,j) for the same proteins. Regions with low BAI are highlighted and the amino acids present in those regions are mentioned.

**Figure 5:**
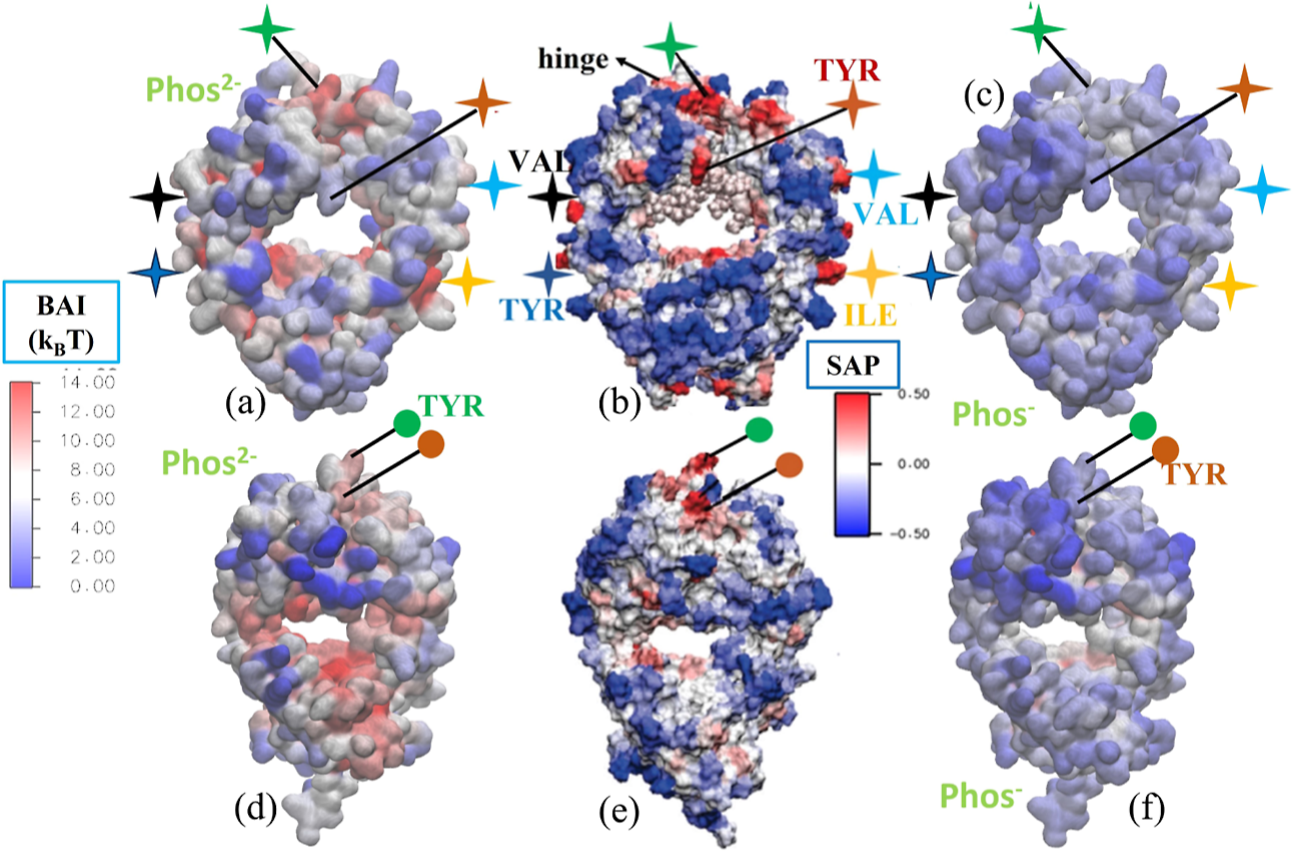
BAI obtained with eq 2 and represented as a color plot on the surface of Fc (top) and Fab (bottom) fragments, for *Phos*^2–^ (a,d) and *Phos*^–^ (c,f) buffer molecules. Lower BAI value (closer to zero) indicates a higher number of contacts between the protein and the buffer, and therefore, a higher adsorption-free energy. We compare the BAI with the SAP color plot (b,e) for the same proteins (results taken from ref (45)). Equivalent regions on the BAI and SAP plots are indicated by stars (for Fc) and circles (for Fab) of the same color.

Figure 4 shows that both *Cit* and *Phos* bind to positively charged regions on the protein surface. Hence, we find a correlation between protein surface charge and adsorption, with regions of higher positive electrostatic potential providing stronger adsorption sites. A comparison of BAI results for *Phos* and *Cit* with *His* (see results in our earlier work^25^) shows that *His* binds more strongly to both charged and hydrophobic regions on the protein surface. We note that the simulations reported by us with the *His* buffer were performed with the same mAb and fragments studied here. We targeted in those simulations a pH = 6, consistent with the experimental conditions used with *His* buffer (see ref (25)). The affinity for the hydrophobic regions is connected to the ability of *His* to engage in different interaction modes (electrostatic, cation−π, h−π, or π–π). Moreover, owing to its zwitterionic nature, the *His* buffer binds to both positively and negatively charged amino acids. Hence, we conclude that *His* features a stronger tendency to block aggregation-prone regions associated with hydrophobic amino acids than either *Cit* or *Phos* buffers. Instead, *Cit* and *Phos* block interaction between positively charged patches on protein surfaces, and therefore, these buffers modify mostly the interprotein electrostatic interaction. We compare in Figure 5 the BAI and spatial aggregation propensity (SAP)^45^ indices, again projected as a color plot on the Fab and Fc surfaces. The regions of low BAI (stronger buffer adsorption) do not correlate with the regions of high SAP calculated in ref (45), which measure the solvent-exposed hydrophobicity on the protein surface. Our results show that both *Phos* and *Cit* buffers do not feature significant adsorption on the aggregation-prone hydrophobic regions on the protein surface. A similar comparison for *His* buffer (see ref (25)) reveals a strong correlation between the high SAP and low BAI regions, indicating significant interaction between *His* and solvent-exposed hydrophobic regions, with *His* blocking these regions effectively.

In Figure S2 of the Supporting Information, we show the normalized probability distribution of BAI for the *Phos* and *Cit* ions and for the Fab and Fc fragments. The most probable BAI is ∼5*k*_B_*T* and ∼7.5*k*_B_*T* for *Phos*^–^ and *Phos*^2–^, respectively. The same trends are observed for both Fab and Fc fragments. However, the higher charged ions feature a higher probability of engaging in strong interactions (BAI → 0) with the protein surface. For the *Cit* buffer, the probability distributions of *Cit*^2–^ and *Cit*^3–^ are close to each other (see, in particular, the Fc results in Figure S2 of the Supporting Information). We also find that the probability for the regions featuring stronger interaction with the buffer (BAI → 0) depends much less on the buffer charge. These results are consistent with the weaker buffer charge dependence found in the rdfs of *Cit* around the Fab and Fc fragments, as reported in Figure 3. The occurrence of the peak at a higher value of BAI indicates that the ions with a higher charge bind very specifically to a small number of amino acids on the surface of the protein, while the lower charge species bind to a larger number of residues through alternative interactions like hydrogen bonding. This is apparent from the BAI color plots (Figure 4) that show a relatively more uniform BAI over the protein surface for the buffer species with a lower negative charge (*Phos*^–^ and *Cit*^2–^). We found in our earlier work on *His* buffer a small difference of 2*k*_B_*T* between *His*^+^ and *His*^0^, with the former showing a maximum at a higher value of BAI.^25^

**Figure 6:**
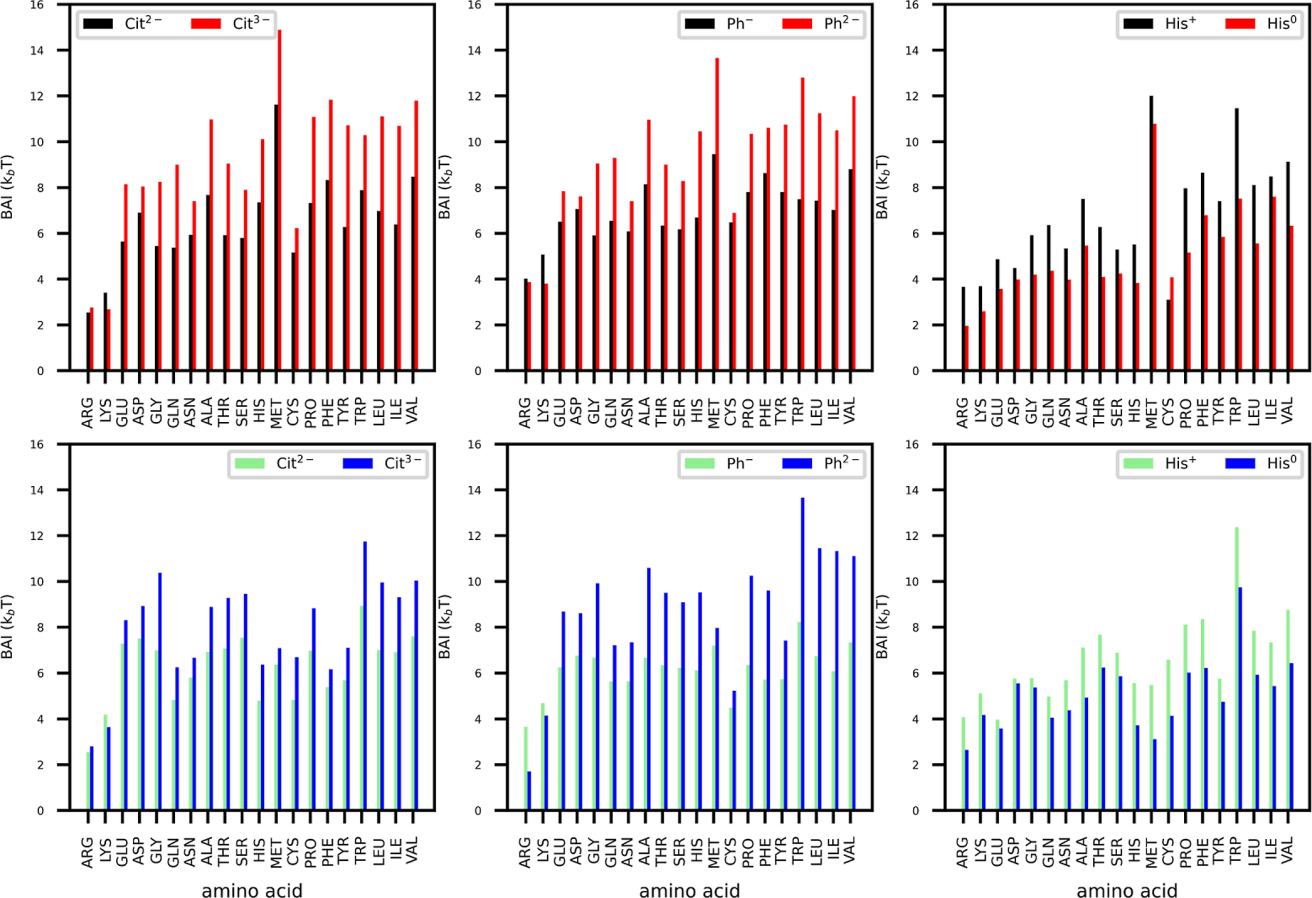
BAI for different surface amino acids in Fab (top) and Fc (bottom) fragments and *Cit*, *Phos*, and *His* buffer systems. The data were obtained by averaging over 5 independent runs for the *Cit* and *Phos* systems, while *His* results have been taken from the simulations performed in ref (25). Results reported for the *His* buffer were performed with the same mAb studied here. Simulations were performed at pH = 6, targeting experimental conditions (see ref (25)).

The BAI color maps show a clear preference for *Phos* and *Cit* to adsorb preferentially at positively charged regions on the protein surface. We have quantified the adsorption at a deeper level by computing the average BAI for different amino acid species (see Figure 6). The average for each species has been calculated over residues that have a non-zero time-averaged solvent accessible surface area (SASA) calculated from the simulations. Both *Phos* and *Cit* adsorb more strongly on the positively charged amino acids, as indicated by the low average BAI values for LYS and ARG (∼2*k*_B_*T*). This trend is reproduced across the three buffers (Figure 6). We expect that the stronger adsorption on ARG as compared to LYS is facilitated by the interplay between the buffer ion and the guanidinium group of ARG, which can form multiple hydrogen bonds with the buffer ions in addition to electrostatic interaction (see Section 2.3 for more details). This observation is in agreement with earlier experimental studies.^46,47^

The comparison of the BAI values for *Phos* and *Cit* with those of *His* (see Figure 6 and ref (25)), supports our idea that the *His* buffer features a stronger preference to adsorb on hydrophobic amino acids, in addition to the charged and polar ones. This idea is supported by the generally lower BAI observed for neutral *His* (*His*^0^) across the whole range of amino acids, as represented in Figure 6. We concluded in ref (25) that the adsorption at hydrophobic spots might provide a mechanism for *His* to inhibit aggregation more efficiently than other buffers, such as *Phos* or *Cit*. The results presented in Figure 6 reinforce this idea.

### 3.3 Protein–Buffer Binding Mechanisms

*Phos*, *Cit*, and *His* are polydentate molecular ions featuring multiple interaction centers. This polydentate nature allows for various different protein-binding modes. These aspects are explored below to identify the protein-buffer binding mechanism.

**Figure 7:**
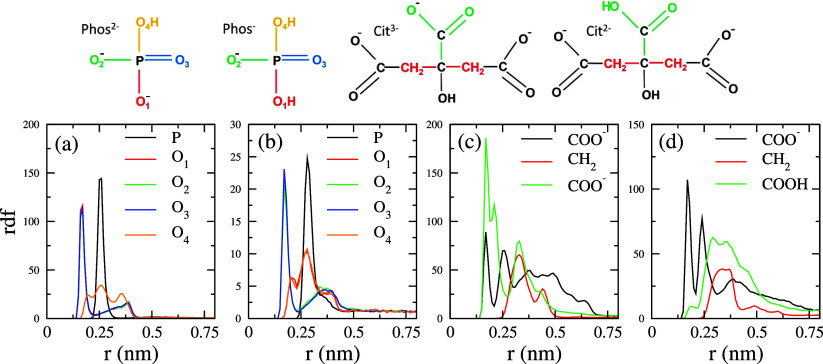
Radial distribution function of different charged groups of the buffer ions around the protein surface. Calculations were performed using the same approach as in Figure 3. Panels show the results for *Phos* interacting with (a) Fab and (b) Fc, and *Cit* interacting with (c) Fab and (d) Fc.

**Figure 8:**
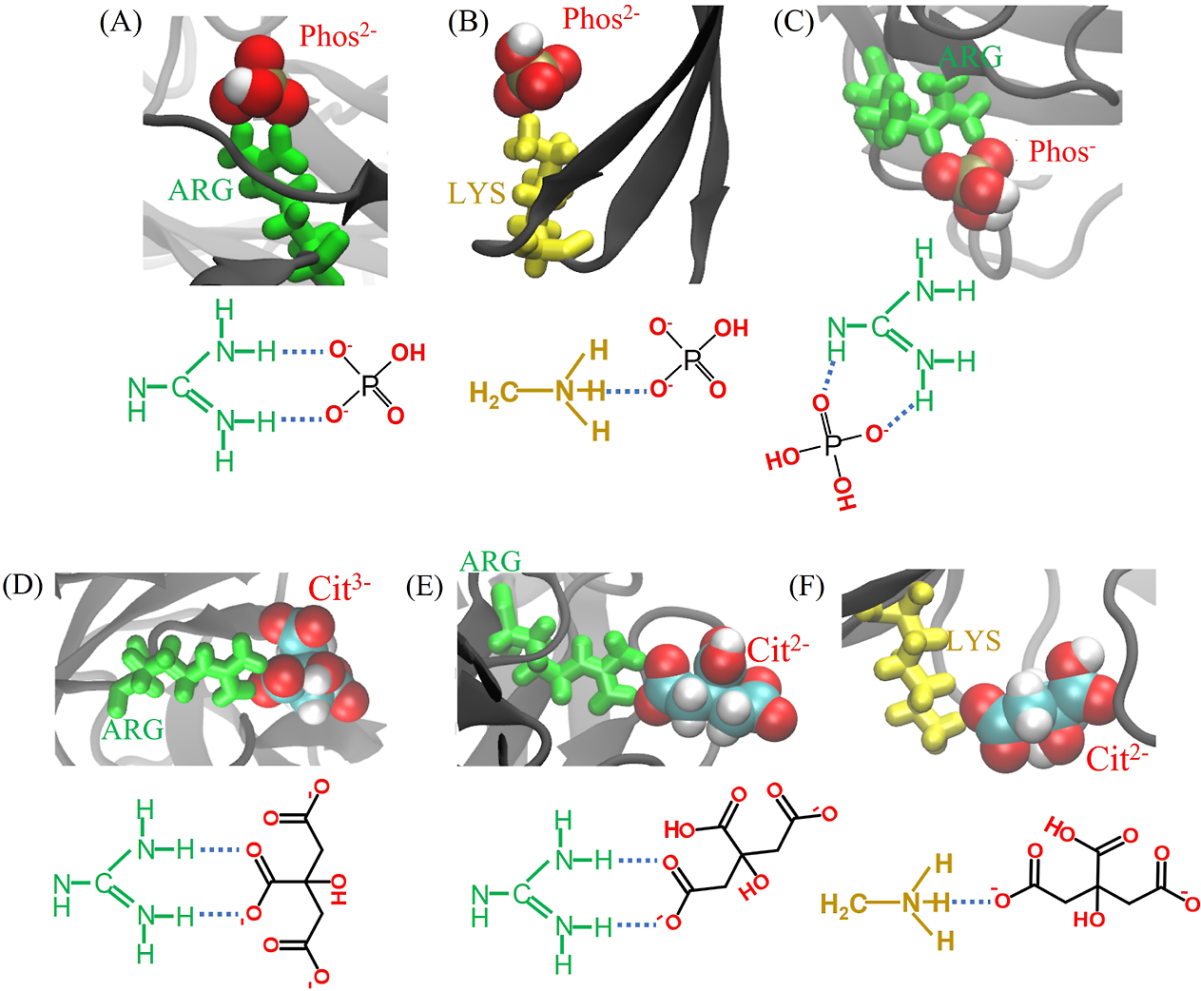
Binding modes of *Phos* and *Cit* ions to the ARG and LYS residues on the Fab or Fc surface. ARG and LYS residues are shown in green and yellow, respectively.

To identify the binding mechanism, we calculated the rdf (unnormalized as in Figure 3, top and middle panels) of different atomic groups of the buffer ions around the Fab and Fc fragments. Figure 7 shows the rdf of *P* and the 4 atoms of *Phos*^2–^ and *Phos*^–^ ions. We also show the rdf of the −COO^–^, −CH_2_, and −COOH groups of the *Cit*^3–^ and *Cit*^2–^ ions. The rdf analysis indicates that the *Phos*^2–^ ion reorients next to the protein, with the O_1_/O_2_/O_3_ atoms in close contact with the protein surface. The O_4_ atom (attached to the hydrogen atom) appears to be more delocalized and lies farther away from the surface. For *Phos*^–^, the O_2_/O_3_ atoms are in direct contact with the protein surface, while the hydrogen-bonded oxygens lie further away (see also the binding modes shown in Figure 8A–C). We conclude that the higher polarity of the O_1,2,3_ in *Phos*^2–^ or O_2,3_ in *Phos*^–^ drives the orientation of the *Phos* ions in contact with the protein surface.

The rdf plots of the *Cit*^3–^ ions show that binding to the protein surface occurs preferentially through the central carboxylic −COO^–^ group. The rdf peak for this group is both more intense and closer to the surface (see also Figure 8D). The resulting ion conformation facilitates stronger interaction of the rest of the two −COO^–^ groups with the positively charged binding sites on the protein surface. Our rdf for these groups has a significantly lower height, indicating that the interaction via lateral −COO^–^ groups is weaker.

The binding modes shown in Figure 8 demonstrate that the buffer ions can engage in more site–site interactions with ARG than with LYS, due to the structure of the guanidinium group of ARG. This observation explains the lower average BAI obtained for ARG (Figure 6).

For *Cit*^2–^ ions, binding occurs preferentially via terminal −COO^–^ (see Figure 8E). This interaction mode biases the orientation of the *Cit*^2–^ ions, and both the central and the other terminal −COO^–^ groups detach from the surface, pointing toward the solution (see rdf peaks appearing at a longer distance >0.25 nm in Figure 7). This binding mode results in the *Cit*^2–^ ion interacting electrostatically with the protein preferentially through one of its functional groups, leaving the other groups free to engage in additional binding, making it possible to act as a bridge between neighboring proteins, as discussed later in Section 3.5.

We demonstrated in ref (25) that the interaction modes of *His* with the protein surface involve a more complex mechanism than the one discussed above for *Phos* and *Cit* ions. For instance, *His* can interact with ARG through electrostatic interaction mediated by its charged COO^–^ terminus and also through π–π interaction between its pentagonal ring and the π cloud of the flat guanidinium group of ARG. The involvement in multiple forms of interaction leads to a compact (curled up) molecular conformation of the *His* buffer near the protein surface (see Figure 9 of ref (25)), which may preclude the formation of *His* bridges between protein surfaces.

**Figure 9:**
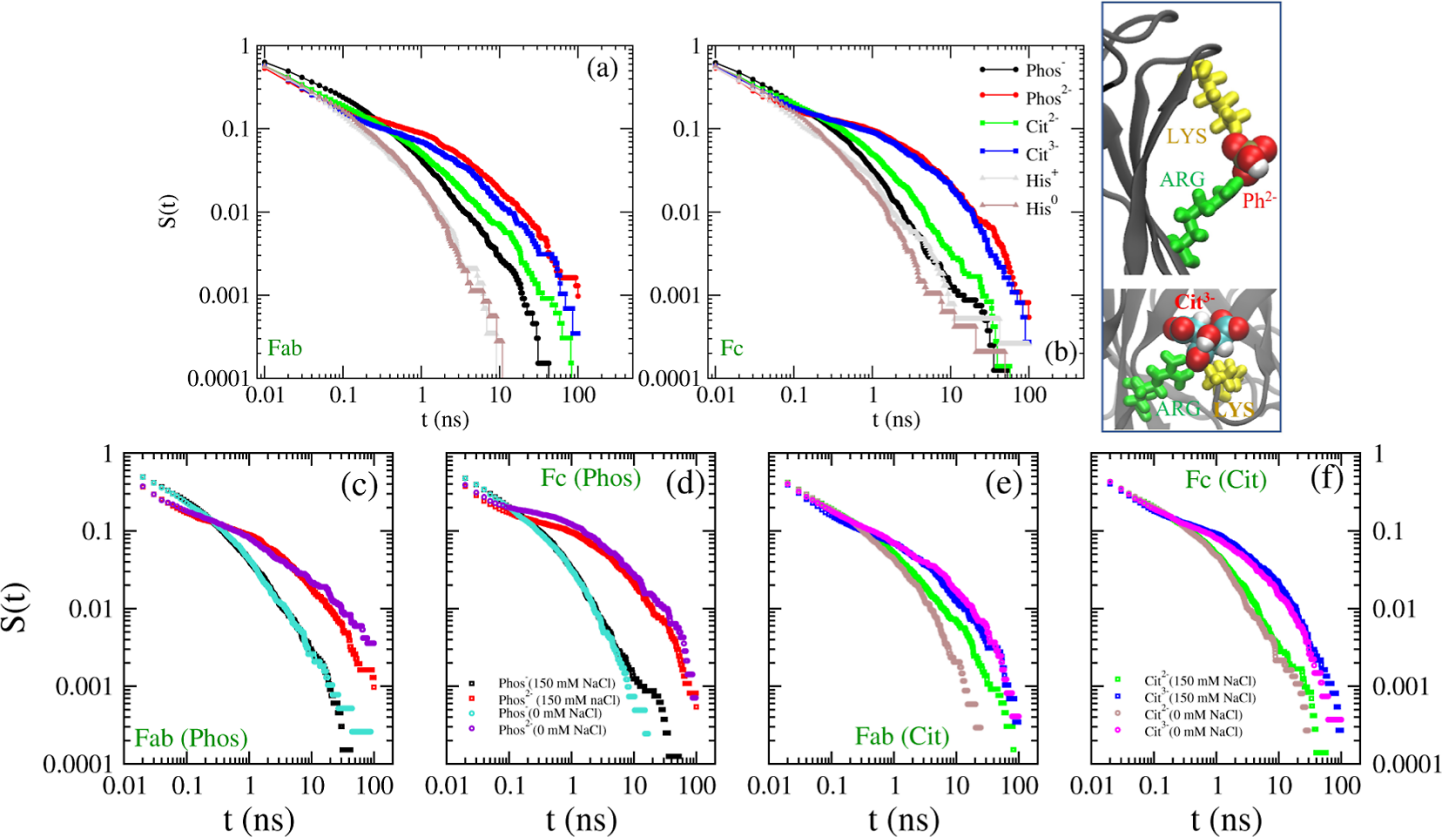
Time dependence of the survival probability (*S*(*t*)) for contacts between (a) Fc, (b) Fab, and the buffer ions. Typical interaction type corresponding to the long-lasting contacts is shown in the panel on the right. The bottom panels show *S*(*t*) in the presence and absence of NaCl for the (c,d) *Phos* buffer, and for the (e,f) *Cit* buffer, for the Fab and Fc fragments.

### 3.4 Buffer Binding Kinetics

The free energy of adsorption of the buffer ions on the protein surface determines the residence time of the ions. The ion-binding kinetics can be quantified via the survival probability of the buffer-protein contacts (see Figure 9 and Figure S3 of the Supporting Information and the associated text for the details of the calculation). Larger survival probabilities spanning longer time scales are correlated with stronger interactions. The computed survival probabilities show that the *Cit* and *Phos* ions form many more long-lasting contacts than *His*. As a reference, for *S*(*t*) = 0.1 (10% survival), *t* = 0.2 ns for *His*,^25^ while for *Cit* and *Phos*, this time scale increases up to ∼0.5 ns.

The analysis of the survival probability indicates that the probability of long-lasting protein contacts with *Cit* and *Phos* increases with ion charge. This observation is consistent with the results presented in Sections 2.2 and 2.3, where we showed that electrostatic interaction dominates the binding mechanism for these ions. In contrast, *His* features very similar survival probability curves for the different charged states (cf. *His*^+^ and *His*^0^ in Figure 9a,b).

For the Fc fragment, with a lower charge (+7*e*, pH = 6) than Fab (+14*e*), the *His* molecules have a larger survival probability than for Fab, with some contacts lasting >10 ns. These long-lasting contacts are observed both in the positively charged *His*^+^ molecule and in neutral *His* (*His*^0^). This result supports a binding mechanism for *His* that is not dominated by Coulombic interaction. Instead, other forms of interaction (e.g., π–π and cation−π) play an important role too (see ref (25)). The ability of *His* to take part in various types of interactions (within a protein) is also known to be important in maintaining the conformational stability of proteins.^48^

Many experiments are performed under added salt conditions, with NaCl being widely used in experimental studies. To understand the impact of salt on the ion-binding kinetics, we performed additional simulations of the Fab and Fc fragments in the presence of *Phos* and *Cit* buffers but in salt-free conditions (see Table 1 for the details of Fab_*Phos*_^7.2^-ns, Fc_*Phos*_^7.2^-ns, Fab_*Cit*_^6^-ns, and Fc_*Cit*_^6^-ns), i.e., in the absence of Na^+^ and Cl^–^ ions. We found relatively minor differences in the survival probabilities obtained with or without salt. Some differences can be observed at long times, but these results might be influenced by statistics, given the limited number of time origins. Hence, we conclude that the salt, NaCl in this case, does not have a significant impact on the ion-binding kinetics of the buffer ions. As survival probabilities are related to the energetics of binding, the salt does not seem to affect the affinity between the protein surface and the buffer ions.

### 3.5 Inter-protein Interaction in the Presence of Buffer

We have shown above that the Coulombic forces dominate the *Cit* and *Phos* interactions with the protein. Here, we examine the role of the buffer, specifically ion-binding effects, on the protein–protein interaction.

We start our discussion by evaluating the electrostatic environment around the proteins in the presence of buffer ions. With this purpose, we define the effective charge (*Z*_eff_)
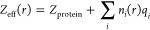
3where *Z*_protein_ is the protein charge, *n*_*i*_(*r*) is the number of ions (*Phos*, *Cit*, Na^+^, or Cl^–^) within a distance *r* of the protein surface, and *q*_*i*_ is its charge. *n*_*i*_(*r*) can be calculated by integrating the rdf’s, shown in Figure 3
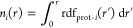
4

**Figure 10:**
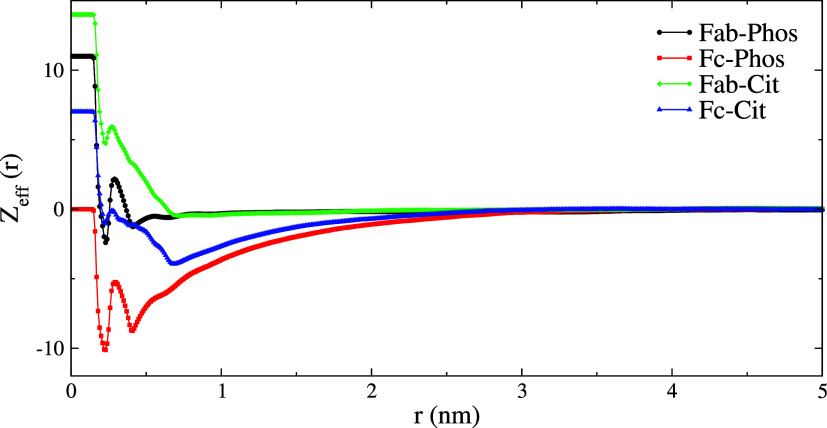
Charge compensation, *Z*_eff_, as a function of distance *r* from the protein surface. Negative regions indicate charge reversal associated with ion adsorption. Results for the *Phos* buffer are obtained at pH = 7.2, while those for the *Cit* buffer at pH = 6, which results in different protein surface (*r* = 0) charges.

We show, in Figure 10, *Z*_eff_ as a function of the distance (*r*) from the protein surface. *Cit* and *Phos* buffers, within a small distance from the protein surface, neutralize the surface charge of the strongly charged Fab fragment. However, for Fc at pH 7.2 (*Z*_protein_ = 0*e*), the adsorption of *Phos* leads to a significant effective charge on the protein, reaching a maximum of ∼−10*e* between *r* = 0 and 0.5 nm. This distance range corresponds to the thickness of the ionic adsorption layer found in Figure 3a–d. The negative effective charge leads to a double layer, evident from the slow decay of the charge with distance (see the red curve in Figure 10). To estimate the screening length for the Fc systems, we fit *Z*_eff_ to an exponential function, *Z*_eff_(*r*) = *a* exp(−*x*/ξ), where ξ represents the screening length (see fitting details in the Supporting Information, Figure S4). The decay lengths are 0.74 and 0.84 nm for the Fc-Cit^6^ and Fc-Phos^7.2^ systems (see Figure 10). These values are similar to the Poisson–Boltzmann result that would be obtained with a NaCl salt concentration of 0.15 mol/L, employed here (see ref (44)). For Fc-Cit^6^ in contact with Fc at pH = 6 (*Z*_protein_ = +7*e*), we find overscreening (see blue curve in Figure 10), with the protein charge overcompensated by *Cit* adsorption. Overscreening is observed in aqueous solutions when the ions adsorb on surfaces with low surface charge. This effect disappears for strongly charged surfaces. More generally, overscreening is driven by interion correlations and observed in aqueous and non-aqueous solutions.^49,50^ Our results are consistent with the fact that overscreening is observed in the protein with a lower charge (Fc, +7*e* at pH 6), while it disappears for the Fab fragment that holds a higher charge (+14*e* at pH = 6). A small minimum indicating overscreening is observed at pH = 7.2 (black curve in Figure 10) in the presence of the *Phos* buffer. This result is consistent with the idea that a higher surface charge leads to less overscreening.

The results indicate that in the presence of *Phos* and *Cit* buffer, the long-range electrostatic repulsion between Fab fragments will be strongly reduced, allowing the proteins to approach each other at closer distances. This effect can potentially modify the stability of protein formulations. However, for Fc, the electrostatic interaction decays more slowly due to the buildup of charge on the neutral (or mildly charged, depending on the pH) Fc protein surface, potentially providing some degree of stability against aggregation. Such behavior would be in line with the phenomenon of reentrant condensation^46,51−54^ observed in experiments, where an initial increase in counterion concentration at a fixed protein concentration leads to protein charge neutralization and cluster formation, while a further increase in counterion concentration leads to redissolution of the clusters resulting from protein charge inversion. In our case, the buffer and salt concentrations are fixed, while native protein charge determines the possibility of charge-inversion or neutralization, determining the nature of interprotein interaction.

We now identify the adsorption mechanisms of the buffer ions confined between two protein surfaces. This analysis is relevant to understanding the role of these ions in mediating protein aggregation. With this aim, we performed simulations with two Fab domains placed close to each other in three different initial relative orientations (Figure 2). Two sets of simulations were performed, 1 at pH = 7.2 with *Phos* buffer (see 2Fab_*Phos*_^7.2^ in Table 1) and the other at pH = 6 with *Cit* buffer (see 2Fab_*Cit*_^6^ in Table 1). In these simulations, we used a higher buffer concentration of 50 mM, to increase the probability of buffer adsorption at the protein–protein interface, with neutralizing Na^+^ ions at 0 mM NaCl in order to eliminate any effect of the ionic strength on the buffer–protein interaction. We also performed similar simulations in the absence of the buffer, keeping the ionic strength constant by replacing each buffer with a charge-equivalent number of Cl^–^ ions. Each *Phos*^2–^ ion, for instance, was replaced by 2 Cl^–^ ions.

**Figure 11:**
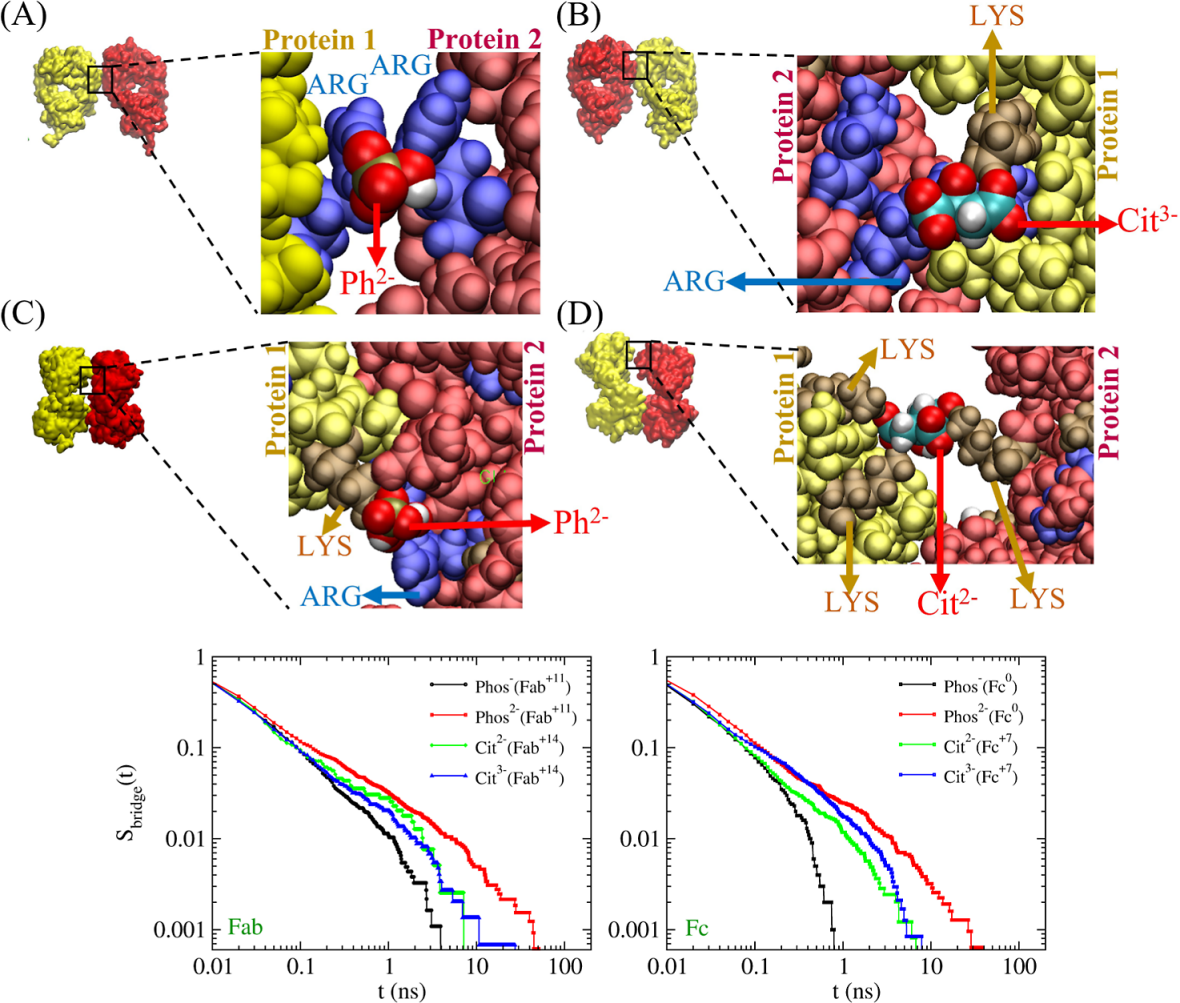
Representative *Phos* and *Cit* bridges between the Fab domains, mediating interprotein interaction. Survival probability of the bridges formed by different buffer species is shown for the 2-Fab and 2-Fc systems (see Figure S10 of Supporting Information for the survival probability of ionic bridges for the Fab–Fc systems).

Our simulations show that *Phos* and *Cit* ions can form bridges between proximate proteins. The bridges are established between positively charged regions on the protein surfaces. The positive charges in Fab and Fc, associated with ARG and LYS (and HIS^+^ at acidic pH) surface residues furnish anchor regions, between which the buffer molecule can form bridges and restrain the inter-protein distance. The ability of the buffer to form bridges is connected to the adsorption modes examined in Figure 8 A–C,E,F, which lead to buffer ion orientations promoting the exposure of dangling groups that protrude into the solution, away from the protein surface. Figure 11 shows snapshots from the 2-Fab simulations showing *Phos* and *Cit* ions mediating the bridging interaction between two Fab domains. Figure 11A shows two ARG residues, one from each Fab, held together by a *Phos*^2–^ ion that interacts simultaneously with both residues. In the absence of *Phos*, we would expect a repulsion between the two proteins. Due to the larger spatial extension of the *Cit* ion, the binding conformations resemble an ionic bridge (see Figure 11D, in particular). Similar bridging interactions mediated by *Cit* ions through simultaneous interaction with ammonium ions adsorbed on yttrium-fluoride (YF_3_) nanoparticle surfaces have been used as a strategy to tune nanoparticle self-assembly.^55^

For the *Phos* anion, the ion conformation at the protein–protein interface and the strong condensation of the positively charged amino acids around the ion (owing to its high charge density) result in strong bonds between neighboring proteins. 2D NMR experiments identified the formation of reversible dimers as the process initiating mAb aggregation.^21^ According to our simulations, the buffer ions could indeed promote the formation of such protein dimers as an early event, leading to more significant aggregate formation. In our simulations, the bridges are mostly formed between Lys–Lys, Lys–Arg, and Arg–Arg pairs. In some cases, surface-exposed His^+^ residues are also present at the bridging sites. These bridges did restrict the orientation of the side chains but did not cause any measurable deformation in the protein structure. To demonstrate this quantitatively, we calculated the radius of gyration (*R*_g_) of the Fab and Fc fragments at pH = 6 from the single fragment simulations (Fc: 2.61 ± 0.04 nm and Fab: 2.49 ± 0.02 nm) and compared with the *R*_g_ of the fragments obtained from Fab–Fc simulations performed at pH = 6 (Fc: 2.58 ± 0.02 nm and Fab: 2.51 ± 0.02 nm). We find that the *R*_g_ values are similar, suggesting no structural change in the proteins due to buffer bridging.

One important variable determining the efficiency of the ion bridges promoting protein dimer formation is the stability of the bridges over time. To quantify the latter, we analyzed the kinetics of formation and dissociation of the buffer-mediated bridges between the Fab fragments by computing the survival probability of the ionic bridges as a function of time. First, we calculated the minimum distance (*d*_min_) between the buffer ions and the protein surfaces for each buffer ion. Then we defined a buffer bridge to exist between the protein surfaces when a buffer ion has a *d*_min_ < 0.4 nm from both protein surfaces. The survival probability for the buffer bridges was calculated using the data on the time for which different bridges stay intact (see Figure 11 and Figure S3 of the Supporting Information and the associated discussion for details). The survival probability data show that the *Phos*^2–^ ions form the most stable bridges between proteins, with bridges lasting as long as ∼50 ns. Hence, both the charge density and molecular structure (especially the polydentate structure) of the buffer ion are important variables determining the bridging efficiency. Similar simulations performed with two Fc fragments also show the *Phos*^2–^ ions forming the most stable bridges between the Fc fragments (see Figure 11).

The simulations involving a pair of Fab proteins were performed by using three independent trajectories with three different starting configurations (see Figure 2). In the run with side 1 of one protein facing side 2 of the other protein (Figure 2), we do not see a significant effect of charge screening or buffer-mediated bridging. In the run with side 2 of both proteins facing each other, a significant amount of bridging is observed, and the fab dimer stays intact for longer. The two faces of the Fab fragment show significant differences in their surface charge (Figure S5 of the Supporting Information), with side 2 featuring a much larger net positive charge. Thus, the bridging interaction seems to be more effective for proteins (or protein regions) with a larger net positive charge.

**Figure 12:**
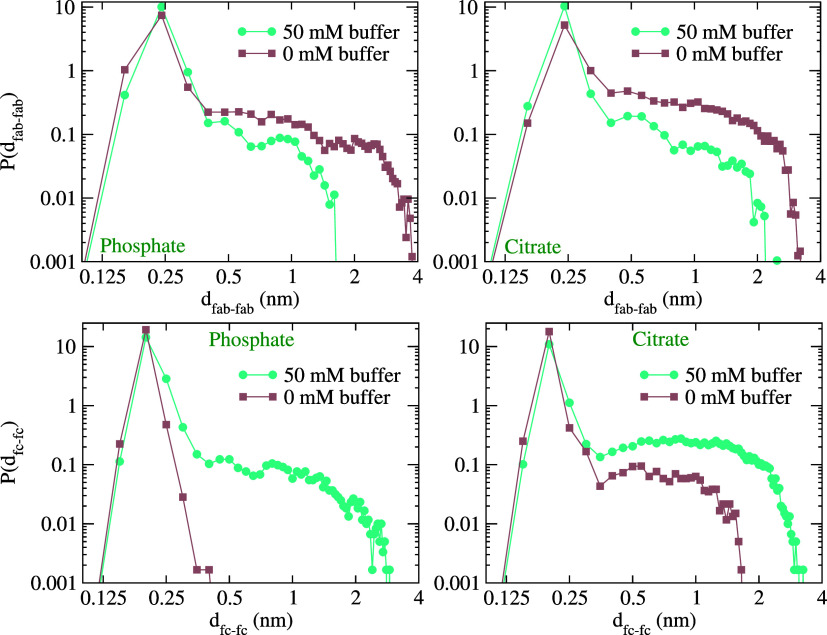
Probability distribution of the minimum distance between the two fragments in the presence (50 mM) and absence (0 mM) of *Cit* and *Phos* buffers obtained from the 2-Fab and 2-Fc simulations, averaged over 3 independent simulations. Separate distributions for the 3 runs are shown in Figures S6 and S7 of the Supporting Information.

We compare in Figure 12, the probability distribution function for two Fab fragments to have a given minimal distance (*d*_fab–fab_; calculated as the minimum of all atomic-pair distances between the two fragments). This probability distribution will feature a maximum at short inter-surface distances if the protein forms a dimer, whereas a flat distribution would indicate that the proteins move freely in solution. The distributions show clear evidence for stable pairs for both *Phos* (pH 7.2) and *Cit* (pH 6) buffers, with maxima at around 0.25 nm interprotein distance. The impact of buffer on the interprotein interaction can be better understood by comparing the probability distributions obtained from simulations in the absence of buffer (where Cl^–^ anions have replaced the *Phos* and *Cit* ions to maintain the ionic strength; see systems 2Fab_*nb*_^7.2^ and 2Fab_*nb*_^6^ in Table 1). In the absence of buffer (0 mM buffer cases in Figure 12), we observe a general increase in the probability of finding longer protein inter-surface distances, indicative of less stable dimers. These results support the role of the buffer ions as bridging units, maintaining the integrity of dimers for longer times. The probability distributions in linear scale and the free energy profiles obtained by inverting the average (over the three independent runs) probability distributions are shown in Figure S6 of the Supporting Information. For *Cit* buffer, the free energy difference between the minimum in free energy (maximum in *P*(*d*_fab–fab_)) and that corresponding to *d*_fab–fab_ = 1.5 nm is ∼7*k*_B_*T* in the presence of buffer, and 3–4*k*_B_*T*, under no buffer conditions (and similar ionic strength). The free energy profiles indicate a stronger effect of the *Cit* buffer ions in stabilizing protein dimers. Given the relevance of dimer formation as a precursor of protein aggregation, we postulate that the bridging mechanism discussed here might be relevant to determining the stability of protein formulations in the presence of *Phos* or *Cit* buffer. Ultimately, our results demonstrate that electrostatic screening coupled with bridging interactions mediated by the buffer ions can have a significant impact on the stability of the protein dimers.

Our analysis of charge compensation (see Figure 10) showed two very different scenarios for Fab and Fc proteins. While Fab features full charge compensation at short distances from the protein surface, Fc-buffer interaction led to the charging of the protein, leading to a slow decay of the charge density spanning ∼2–3 nm from the protein surface. Based on these results, we might predict a different behavior for the dependence of the Fc dimer stability with buffer. To address this point, we performed additional simulations of Fc dimers (systems 2Fc_*Phos*_^7.2^ and 2Fc_*Cit*_^6^ in Table 1) and explored the stability as a function of the minimum interprotein distance (see Figure 12). For Fc, the probability distribution for the distance of closest approach between the Fc fragments (*d*_fc–fc_) is broader in the presence of the buffer ions, whereas, in the absence of the buffer ions (buffer ions replaced by Cl^–^ ions; systems 2Fc_*nb*_^7.2^ and 2Fc_*nb*_^6^ in Table 1), the Fc fragments stay close to each other, as shown by the probability distributions (see also Figure S7 of Supporting Information), which are narrow and attain maxima at short interprotein distances. This result is consistent with our earlier studies showing that Fc fragments feature negative second virial coefficients at a salt concentration of 150 mM, and in the absence of buffer.^44^ However, the behavior is opposite to that observed for the Fab fragments. Indeed, in the presence of *Phos* buffer, the probability distribution for *d*_fc–fc_ changes significantly and shows a higher probability for larger values, indicating the dissociation of the Fc-dimers. This result shows that the charge reversal associated with the buffer–protein interaction (see Figure 10) reverses the interprotein interaction, which becomes repulsive for the Fc fragments in the presence of buffer.

We infer from our computations that the impact of the *Phos* and *Cit* buffers on protein aggregation is dependent on the protein charge. For the Fab fragment with a higher positive charge, the presence of the *Phos* and *Cit* buffers leads to an increase in the level of dimer formation through a combined effect of charge screening and buffer-mediated bridging between the proteins. However, for the Fc fragment, which bears a lower positive charge, the presence of the buffer leads to a higher repulsion than that in the presence of an equivalent amount of monovalent ions. The repulsion emerges from the charge-reversal of the protein surface in the presence of the buffer.

To understand the impact of buffer on the Fab–Fc interaction, we performed additional simulations of one Fab and one Fc fragment placed in the simulation box in the presence and absence of buffer (see systems (Fab–Fc)_*Phos*_^7.2^, (Fab–Fc)_*Cit*_^6^, (Fab–Fc)_*nb*_^7.2^, and (Fab–Fc)_*nb*_^6^ in Table 1) with relative starting conformations similar to those employed for the 2-Fab and 2-Fc systems (see Materials and Methods). Interestingly, for this system, we obtained contrasting results for the *Phos* and *Cit* buffers (see Figure S8 of Supporting Information). While the dimer stability is similar in the presence of the *Phos* buffer (see free energy corresponding to pH = 7.2 in Figure S8), the dimer stability in the presence of *Cit* buffer is lower (see increase in free energy at *d*_fab–fc_ = 1.5 relative to the no buffer system in Figure S9 of Supporting Information). This result might highlight the importance of charge reversal observed in the Fc fragment in the presence of both *Phos* and *Cit*. For the specific case of *Cit*, we find charge reversal and evidence for slow decay in the electrostatic potential (see the blue line in Figure 10). This buildup of negative charge might interact with the *Cit* ions around the Fab fragment, leading ultimately to an enhancement of the Fab–Fc repulsion and dimer stabilization, as reported in Figure S9.

## 4 Conclusions

We have investigated, using all-atom MD simulations, the interaction between Fab and Fc protein fragments of mAB COE3 and buffer ions commonly used in formulations: *Cit*, *Phos*, and *His*.

Our simulations reveal significant differences in the interaction mechanisms of the different buffers with the surface of the protein. *His* binds by blocking hydrophobic regions on the Fab/Fc surface, in addition to binding to charged regions. *Phos* and *Cit* ions bind preferentially to the positively charged regions on the protein surface. We rationalize the different binding behaviors in terms of the higher charge densities of the *Phos* and *Cit*. The stronger electrostatic interaction of the *Phos* and *Cit* ions with Fc and Fab fragments is reflected in a significant increase in the adsorbed ion residence time relative to *His*.

The strong electrostatic interaction of the *Cit* and *Phos* ions with the protein surface leads to a significant buildup of charge in the Fc fragment. At pH 7.2 conditions, Fc has zero charge, but the adsorbed *Phos* ions form a shell of negative charge on the protein surface, leading to a significant surface charge density corresponding to ∼−10*e*. At pH 6, Fc has a net charge of +7*e*, and the *Cit* ions overscreen the Fc charge, resulting in a charge reversal at the protein surface. The relationship between buffer and aggregation might be closely connected to the buffer-protein electrostatic interaction mechanism. The neutralization of the surface charge and full screening at high protein charges would favor more aggregation due to the inhibition of double-layer repulsion. Instead, a mechanism leading to a buildup of charge on a neutral protein might lead to double-layer repulsion that could stabilize the protein solution against aggregation. Our simulations support this notion.

We have uncovered a mechanism of the buffer-protein interaction that might be relevant to understanding the stability of protein formulations. The mechanism is closely connected to the protein charge state as well as the preferential interaction of *Phos* and *Cit* with the protein surface. We demonstrate that both *Phos* and *Cit* ions act as bridges between protein surfaces, following a complex ion reorientation mechanism that depends on the ionic charge. The ionic bridges consist of buffer ions simultaneously interacting with the ARG and LYS residues on the surfaces of the two different proteins. This bridging mechanism could potentially increase the lifetime of encounter complexes between natively folded proteins, possibly influencing the aggregation under conditions relevant to therapeutic formulations. Indeed, recent kinetic models for protein aggregation assume the formation of irreversible dimers as an integral part of the protein aggregation pathway.^19^ Buffer-mediated increase in the lifetimes of these dimers could accelerate protein unfolding and consequently enhance aggregation rates. We conclude that this mechanism is prevalent in Fab. In this case, the high protein charge at standard pH conditions and the protein–buffer interaction result in full compensation of the protein charge at a short distance from the protein surface. Our analysis of the Fc fragment depicts a very different scenario. In the absence of a buffer, the Fc–Fc interaction is predominantly attractive. However, the buildup of charge at the protein surface, emerging from the buffer–protein interaction, leads to an effective interprotein repulsion that destabilizes Fc-dimer formation. Hence, we expect that the aggregation between mAbs in the presence of buffer might be initiated through the Fab–Fab fragment interaction.

We believe that the dependence of the aggregation pathway on native protein charge might play a role in a wide range of protein solutions. As discussed earlier, the dissolution at a high salt concentration of aggregates formed at an intermediate salt concentration constitutes the phenomenon of reentrant condensation. The dissolution is associated with the reversal of the protein surface charge due to ion condensation on the protein surface. In our case, we also find that the variation in interprotein interaction in the presence of buffer (from attractive in the case of Fab to repulsive in the case of Fc) is a function of an interplay between native protein charge (determined by the pH) and the protein surface charge associated with ion adsorption.

Like reentrant condensation, which is seen for a wide range of proteins, starting from ovalbumin, β-lactoglobulin, and lysozyme^52^ to disordered proteins^46^ and monoclonal antibodies,^56^ we expect the abovementioned dependence of the aggregation pathway on native protein charge to be relevant to a wide range of mAbs belonging to the IgG family, like the one studied in our work (COE3).

We anticipate that the results presented in this work will contribute to refining and developing aggregation kinetic models that have proven to be helpful in modeling the aggregation of monoclonal antibodies. The development of such models will help in the design of more effective and stable therapeutic formulations.

## Supporting Information Available

p*K*_a_ values for *Phos*, *Cit*, and *His*, composition of different simulation systems, distribution of BAI for different buffer species and charge states, details of survival probability calculation, debye length calculation from effective charge as a function of the distance from protein surface, distribution of charged amino acids on the Fab surface, probability distribution of *d*_fab–fab_, *d*_fc–fc_, and *d*_fab–fc_ in the log and linear scales for each run with the associated free energy profiles, and the survival probability of ionic bridges for the Fab–Fc systems (PDF)
