# Supplementary material for: Unraveling the Microscopic Mechanism of Molecular Ion Interaction with Monoclonal Antibodies: Impact on Protein Aggregation
Source: Mol Pharm. 2024 Feb 12;21(3):1285–99. doi: 10.1021/acs.molpharmaceut.3c00963 (PMC10915798; doi:10.1021/acs.molpharmaceut.3c00963)
Supplement: Supplementary file 1 — mp3c00963_si_001.pdf [file mp3c00963_si_001.pdf]

Unravelling the microscopic mechanism of molecular ion interaction with monoclonal antibodies: Impact on protein aggregation (Supplementary Information)

Suman Saurabh,[†] Qinkun Zhang,[†] John M. Seddon,[†] Jian R. Lu,[‡] Cavan Kalonia,[¶] and Fernando Bresme[*,†]

†*Department of Chemistry, Molecular Sciences Research Hub, Imperial College, W12 0BZ, London, United Kingdom*

‡*Biological Physics Group, School of Physics and Astronomy, Faculty of Science and Engineering, Oxford Road, The University of Manchester, Manchester M13 9PL, UK*

¶*Dosage Form Design and Development, BioPharmaceutical Development, BioPharmaceuticals R&D, AstraZeneca, Gaithersburg, Maryland 20878, United States*

E-mail: f.bresme@imperial.ac.uk

Phone: +44 207 594 5886

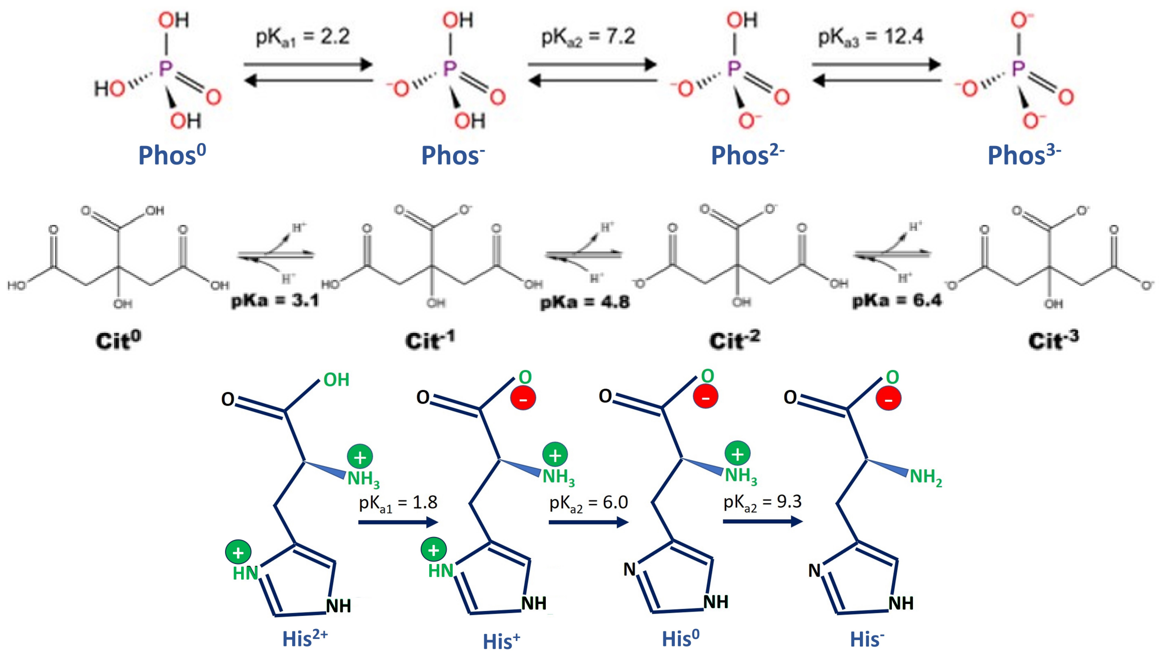

Figure S1: pK_a values for the phosphate (top), citrate (middle) and histidine (bottom) molecules.

Table S1: The number of buffer ions, NaCl and water molecules for different systems simulated in this work (see table 1 in the main text). The variability in the number of water molecules stems from different initial position of the buffer ions in the different copies of the systems. The difference is smaller for the *Phos* systems due to their smaller molecular size.

System	System name	$N_{Phos^-}/N_{Phos^{2-}}$	$N_{Cit^{2-}}/N_{Cit^{3-}}$	N_{Na^+}/N_{Cl^-}	N_{wat}
1.	$Fab^{7.2}_{Phos}$	10/10	–	167/148	54095–7
2.	$Fc^{7.2}_{Phos}$	10/10	–	178/148	53962–70
3.	$Fab^{7.2}_{Phos}$-ns	10/10	–	19/0	54391
4.	$Fc^{7.2}_{Phos}$-ns	10/10	–	30/0	54266
5.	Fab^{6}_{Cit}	–	12/8	182/148	54035–55
6.	Fc^{6}_{Cit}	–	12/8	189/148	53889–900
7.	Fab^{6}_{Cit}-ns	–	12/8	34/0	54331–51
8.	Fc^{6}_{Cit}-ns	–	12/8	41/0	54185–95
9.	$2Fab^{6}_{Cit}$	–	30/20	92/0	51964–2001
10.	$2Fab^{7.2}_{Phos}$	25/25	–	53/0	52147–213
11.	$2Fab^{6}_{nb}$	–	–	92/120	52241–96
12.	$2Fab^{7.2}_{nb}$	–	–	53/75	52326–79
13.	$2Fc^{6}_{Cit}$	–	30/20	106/0	51692–759
14.	$2Fc^{7.2}_{Phos}$	25/25	–	75/0	51903–45
15.	$2Fc^{6}_{nb}$	–	–	106/120	51981–2043
16.	$2Fc^{7.2}_{nb}$	–	–	75/75	52067–119
17.	$(Fab\text{-}Fc)^{6}_{Cit}$	–	30/20	99/0	51802–21
18.	$(Fab\text{-}Fc)^{7.2}_{Phos}$	25/25	–	64/0	52008–33
19.	$(Fab\text{-}Fc)^{6}_{nb}$	–	–	99/120	52095–115
20.	$(Fab\text{-}Fc)^{7.2}_{nb}$	–	–	64/75	52175–203

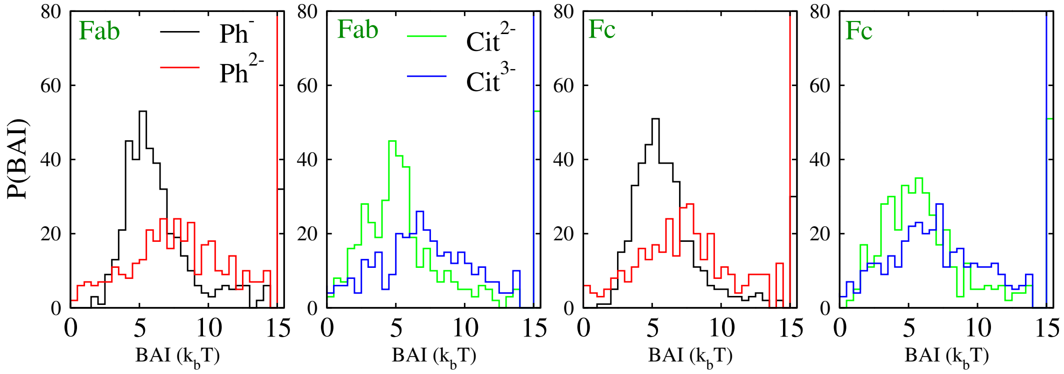

Figure S2: Distribution of BAI values.

Calculation of survival probability

We calculated the time-series of the shortest of all atomic-pair distances, d_{min}, between each buffer molecule and the protein form our MD trajctories. d_{min} defines the separation between a buffer molecule and the protein surface. A buffer molecule was deemed to be adsorbed on the protein if $d_{min} \leq 0.4$ nm. The cutoff was set such that both hydrogen bonds (cutoff acceptor-hydrogen distance of ∼0.25 nm) and salt-bridges (cutoff distance of ∼0.4 nm) are included (D Xu, CJ Tsai and R Dussinov, *Protein Eng. 1997, 10, 999–1012*).

The time-series of d_{min} was calculated for each buffer molecule. We show in Figure S3 the variation of d_{min} with time for a single Cit^{3-} buffer molecule as an example. The trajectory can be decomposed into a series of intervals: time regions where the d_{min} for a buffer molecule either lies within or beyond a distance of r_{cut} from the protein surface. The stretch of time for which $d_{min} \leq r_{cut}$ corresponds to a *residence event* and the time interval is called the *residence time* (τ_r).

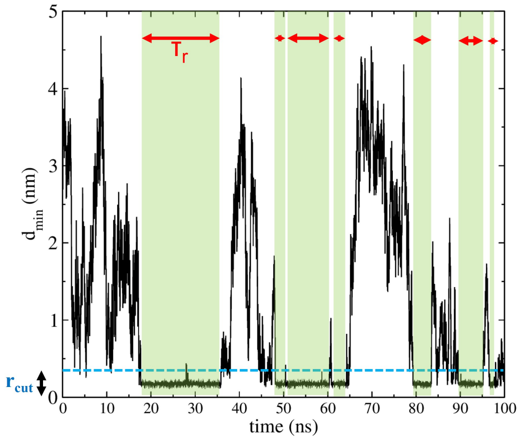

Figure S3: Time dependence of the minimum distance, d_{min}, of a Cit^{3-} buffer molecule from the Fc surface. The regions of the trajectory where d_{min} is below $r_{cut} = 0.4$ nm correspond to adsorption events (shaded in blue) while the rest of the trajectory corresponds to free diffusion of the buffer molecule in solution. The length of an adsorption event τ_r defines the residence time of the buffer for that event.

The time dependence of d_{min} for all buffer molecules of a given type and charge state was

used to study the adsorption kinetics by calculating the survival probability $S(t)$,

$$S(t') = \frac{\langle h(0)h(t') \rangle}{\langle h \rangle} \tag{1}$$

Here, $h(0) = 1$ if a buffer-protein contact is present at time $t = 0$ and 0 otherwise, while $h(t')$ is 1 if a contact, present at t=0, is still intact at time $t = t'$. If re-attachment takes place due to diffusion of a given buffer molecule back from the solution, we consider this event as a new adsorption event. $S(t)$ can thus be defined as the probability that a buffer-protein contact that exists at time 0, continues to exist at least up to time t. As $S(t)$ depends on the strength of buffer-protein interaction, a comparison provides information on the relative affinities of different buffer species and charge states towards the protein surface.

For calculating the survival probability of buffer-mediated bridges between protein surfaces, a buffer molecule was said to be bridging the protein surfaces if it had a $d_{min} < 0.4$ nm for both protein surfaces simultaneously. This criterion was then used for the calculation of residence times and survival probabilities as discussed above.

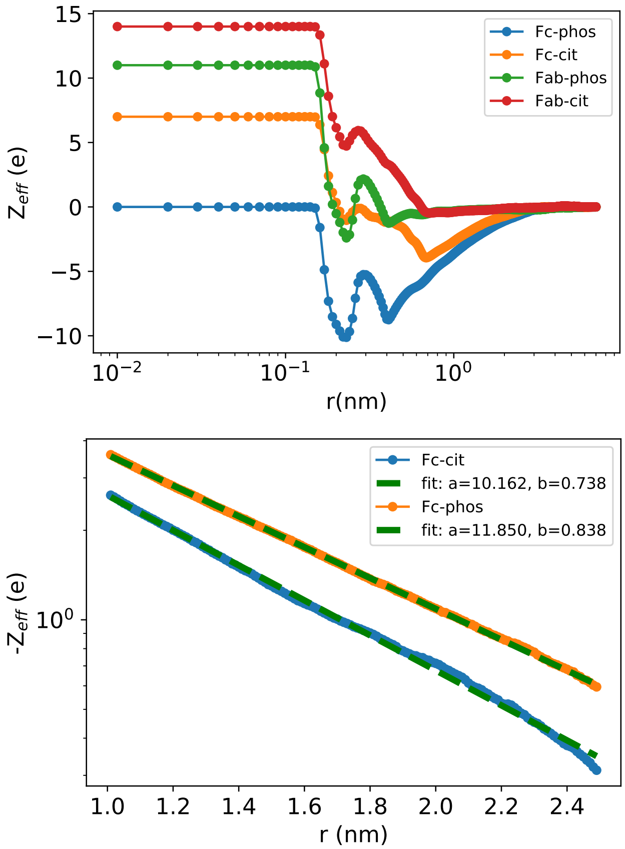

Figure S4: (Top) Compensation of the protein charge as a function of the distance, r, to the protein surface. (Bottom) Fitting of the Fc-cit and Fc-phos to an exponential decay ($a \cdot exp(-r/b)$). The fitting was performed using the data in the region 1-2.5 nm, where the charge $Z_{eff}(r)$ varies exponentially. The number in the legend shows the fitting parameters. with b, being the Debye length, ξ.

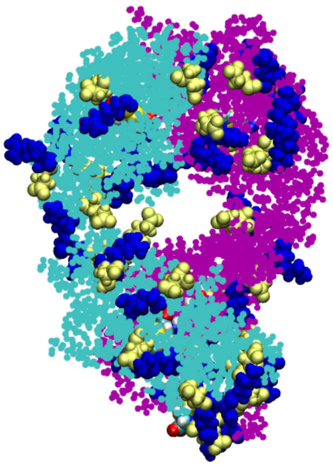

(A) Side 1

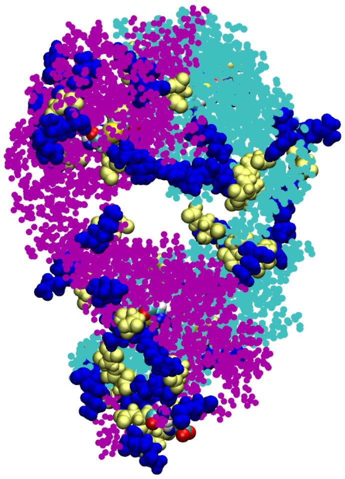

(B) Side 2

Figure S5: The difference in the charged amino acid composition if the two sides of the Fab fragment of mAb COE3. The left figure shows side 1 which has a good balance of the positively and negatively charged amino acids shown in blue and yellow respectively. The right figure corresponds to side 2 which has a majority of positively charged amino acids leading to a higher net positive charge.

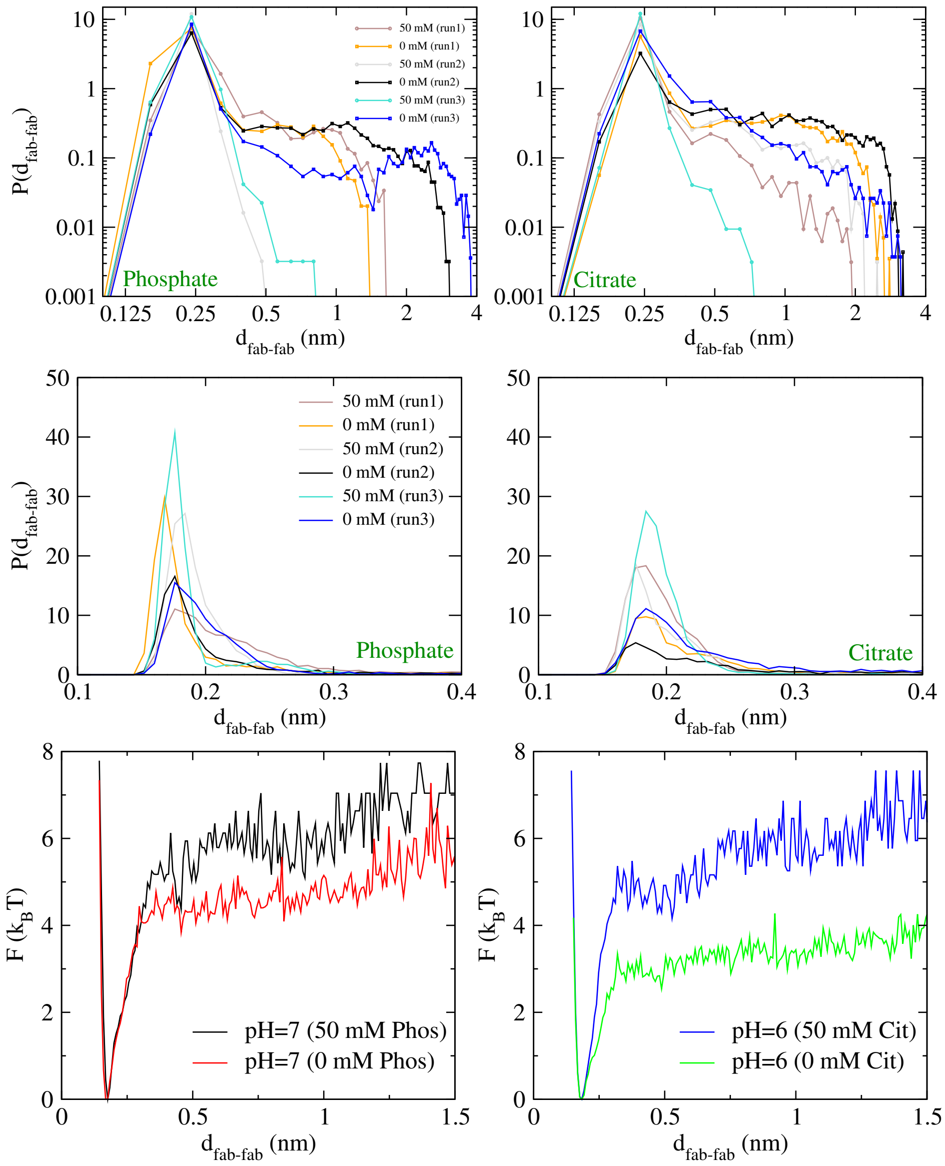

Figure S6: (Upper panel) The probability distribution for $d_{fab-fab}$ for different 2-fab systems simulated in this work, shown for 3 independent runs in the logarithmic scale. (Middle panel) The probability distributions shown in the linear scale. (Lower panel) The average probability distributions over the 3 runs in the presence and absence of buffer have been inverted to obtain the free energy profiles as a function of $d_{fab-fab}$.

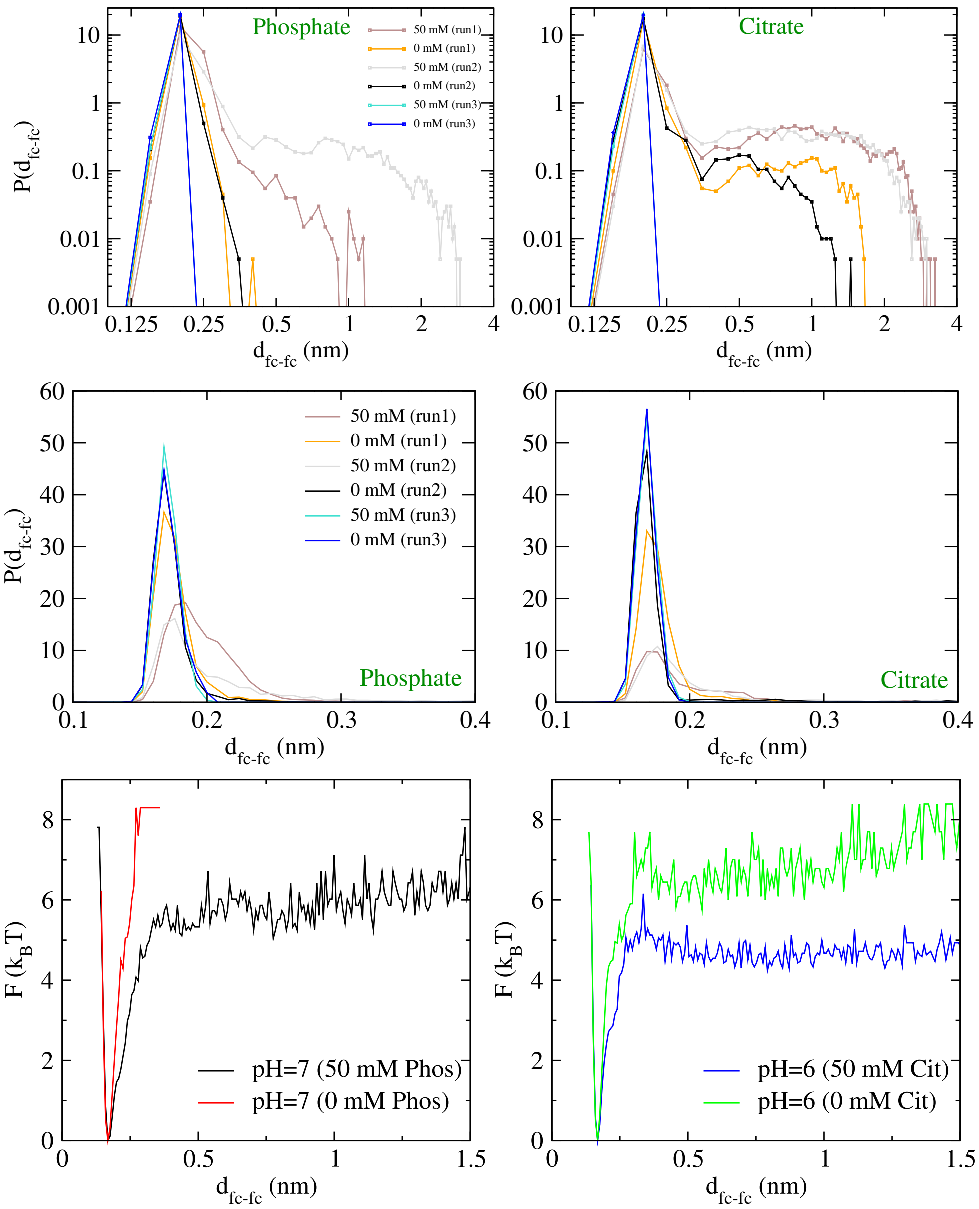

Figure S7: (Upper panel) The probability distribution for d_{fc-fc} for different 2-fab systems simulated in this work, shown for 3 independent runs in the logarithmic scale. (Middle panel) The probability distributions shown in the linear scale. (Lower panel) The average probability distributions over the 3 runs in the presence and absence of buffer have been inverted to obtain the free energy profiles as a function of d_{fc-fc}.

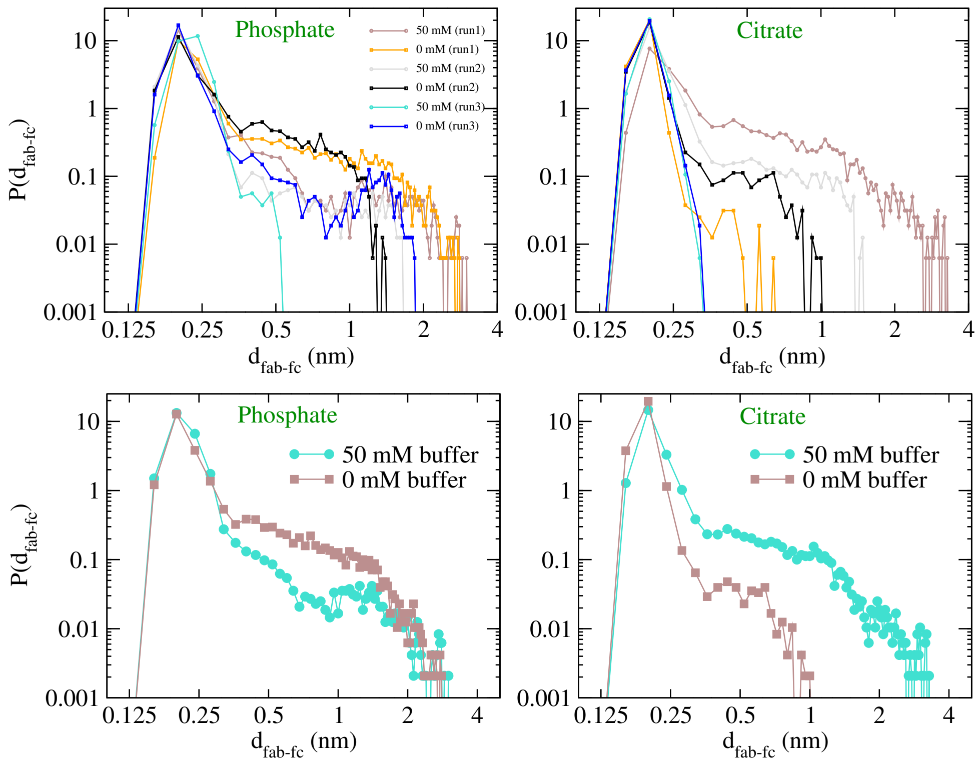

Figure S8: (Upper panel) Probability distribution of the minimum distance between the Fab and Fc fragments in the presence (50 mM) and absence (0 mM) of citrate and phosphate buffers obtained from the Fab-Fc simulations, for 3 independent runs, in the logarithmic scale. (Lower panels) Average of the distributions over the three independent runs.

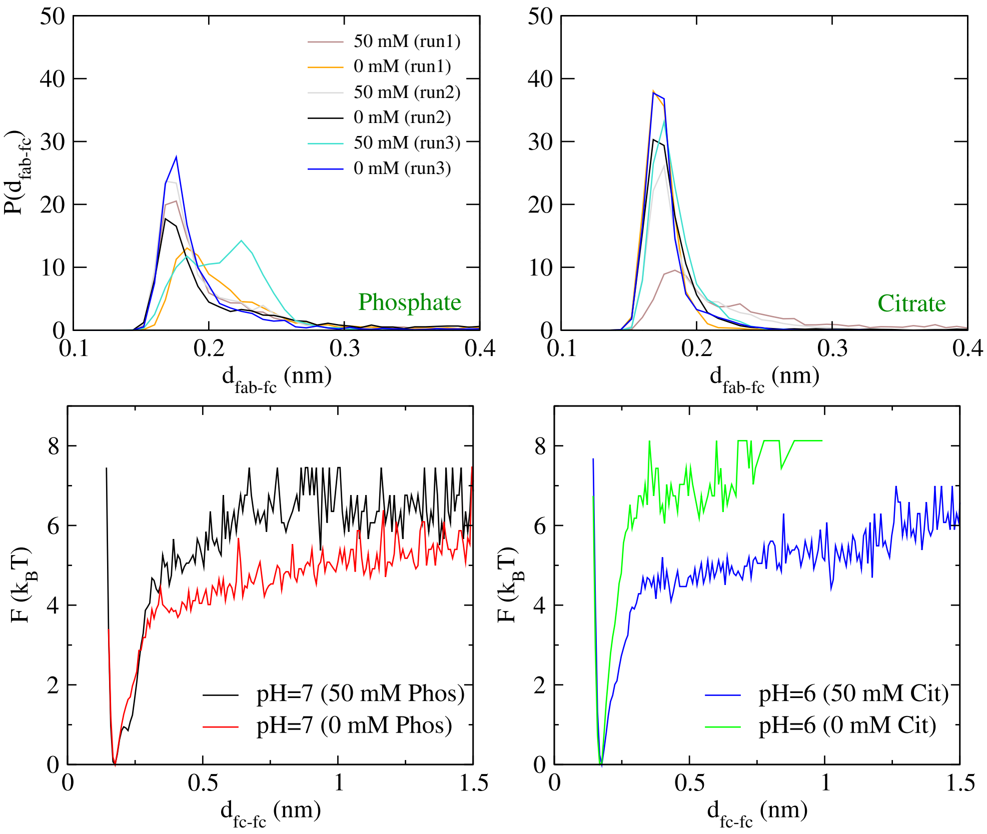

Figure S9: (Top panel) The probability distribution for d_{fab-fc} for different Fab-Fc systems simulated in this work. (Lower panel) The average probability distributions over the 3 runs in the presence and absence of buffer have been inverted to obtain the free energy profiles as a function of d_{fab-fc}.

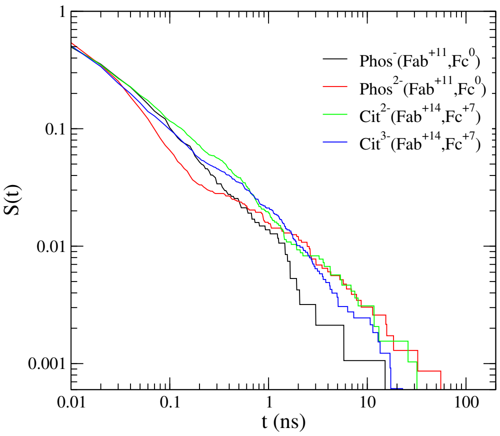

Figure S10: The survival probability of the bridges formed by different buffer species is shown for the Fab-Fc system.
